# Recent progress of carbon dots in targeted bioimaging and cancer therapy

**DOI:** 10.7150/thno.70721

**Published:** 2022-03-14

**Authors:** Cheng-Long Shen, Hang-Rui Liu, Qing Lou, Feng Wang, Kai-Kai Liu, Lin Dong, Chong-Xin Shan

**Affiliations:** 1 Laboratory of Materials Physics, Ministry of Education, and School of Physics and Microelectronics, Zhengzhou University, Zhengzhou 450052, China.; 2 Department of Oncology, The First Affiliated Hospital of Zhengzhou University, Zhengzhou, 450052, China.

**Keywords:** Carbon dots, targeted bioimaging, cancer therapy, nanotheranostic

## Abstract

Carbon dots (CDs), as one new class of carbon nanomaterials with various structure and extraordinary physicochemical properties, have attracted tremendous interest for their potential applications in tumor theranostics, especially in targeted bioimaging and therapy. In these areas, CDs and its derivatives have been employed as highly efficient imaging agent for photoluminescence bioimaging of tumors cells. With unique structure, optical and/or dose attention properties, CDs have been harnessed in various nanotheranostic strategies for diverse tumors through integrating with other functional nanoparticles or utilizing their inherent physical properties. Up to now, CDs have been approved as novel biomaterials by their excellent performances in precise targeted bioimaging and therapy for tumors. Herein, the latest progress in the development of CDs in targeted bioimaging and tumor therapy are reviewed. Meanwhile, the challenges and future prospects of the application of CDs in promising nanotheranostic strategies are discussed and proposed.

## 1. Introduction

Cancer has been one of the widely spread diseases around the world, leading to millions of deaths every year 1-4. Up to now, quite a few research has been carried out on the causes and biology of cancer, dramatically accelerating the development of diagnosis and treatment strategies of cancers 3, 5-12. In the areas of cancer diagnosis, targeted imaging for tumor cells is considered as a promising technology to *in vivo* distinguishing the tumor sites efficiently and precisely, proving the promising diagnosis of cancer without trauma and expensive cost in common detailed physical examination 13-18. Meanwhile, the *in vivo* imaging also provides the ability to monitor tumor morphologies in real-time, allowing doctors to understand the evolution of tumor tissues and enabling to adjust the dosing of drug to abate overtreatment of harmful side-effects, or undertreatment of incomplete cancer remission 13-15, 17-28. Yet, diverse models of targeted imaging have been established with the accumulation biodistribution of imaging agent in tumor sites 17-18, 26-28. However, there are always various limitations for the common imaging agent, such as the inability to bypass biological barriers, poor biocompatibility, lack of stability, etc. In the areas of cancer therapy, there are various recommended guidelines of clinical oncology treatment for different kinds of tumors, such as operative treatment, chemotherapy, radiation therapy, etc. 10, 29-32. Nevertheless, the common strategies for cancer therapy also still remind diverse restrictions such as serious damages to living body, potential drug resistance, ineffectiveness against metastatic disease, drug resistance of cancers, and lack of effective modality for treatment monitoring. With the development of advanced technologies, the concept of precise targeted therapy for tumors are proposed, enabling the ability to overcome the limitations in common strategies of cancer therapy 17, 28, 33-36

In recent years, the excellent performance of nanotheranostic strategies for diverse cancer has been approved with various experiments, enabling the engineered imaging agent or medicines to overcome the specific limitations of some cancers 37-41. Up to now, several nanotheranostic strategies through targeted bioimaging and therapy have been designed to improve the targeting or therapies of cancers 42-46. For examples, some nanomaterials, such as Au nanoparticles (Au NPs), semiconductor polymer nanoparticles (SPNPs), graphene and superparamagnetic iron oxide (SPIO), have been approved for targeted bioimaging with different models, such as photoluminescence (PL), afterglow, chemiluminescence (CL), photoacoustic (PA), and magnetism imaging and precise therapy through targeted drug delivery or advanced biological treatment 47-54. Additionally, several nanomedicines have been approved and recommended as guidelines of clinical oncology treatment for several specific cancers 33, 35. While these nanotheranostic strategies are complex and restricted in clinical treatment, their potential utility has substantiated the investment required at the front-end 27, 55-60. Drawing back on these nanotheranostics strategies, the advanced nanotechnologies of targeted bioimaging and therapy for tumors have been utilized and developed with various approaches, enabling their advantages of efferent and precisely therapeutic effect.

Since the discovery in the process of electrophoretic purification of single walled carbon nanotubes in 2004, fluorescent carbon dots (CDs) have been proved one new class of carbon-related nanomaterials owing to their extraordinary physicochemical properties and diverse potential applications 61-83. With different approaches to preparation, the obtained CDs can illustrate wide diversity in sizes, structures, surface function group and physicochemical properties 78-91. Typically, CDs are always classified as one kind of 0 D carbon nanomaterials, which is consisted of sp^2^/sp^3^ carbon skeleton and abundant functional groups/polymer chains 87-101. Compared with the other bio-nanomaterials, such as phosphorene, 2D Xenes, hydrogels, hydrophobic organic polymers, semiconducting polymer nanoparticles, the CDs, as one zero-dimensional carbon-based nanomaterials, have illustrated obvious advantages in structure and properties for the application of nanotheranostics: (I) CDs can achieve different luminescent models, such as PL, phosphorescence, thermally activated delayed fluorescence (TADF), chemiluminiscence, etc., which can be used for diverse models of bioimaging; (II) CDs behave excellent biocompatibility and photostability compared with other imaging probes, which is favorable to be used as *in vivo* real-time imaging agents; (III) CDs exhibit tunable PL emission, especially the near-infrared (*NIR*) emission, which is suitable for tissue imaging with deep penetration; (IV) CDs are proved unique properties, such as penetration of blood brain barrier (BBB) and novel targeting for some cells, which is extraordinary advantages for imaging; (V) CDs exhibit clearable property in living body, which is excellent for long-term imaging. In addition to the applications as imaging agent, CDs have been approved to be as a nanoplatform to design biomaterials or directly used as nanomedicines for treatment of several special kinds of diseases 102-107. On the one hand, the CDs possess a sp^2^ hybridized structure with different surface functional groups, enabling their novel physical properties including excellent water solubility, biocompatibility, and photophysicochemical properties. Meanwhile, due to their special structure, some CDs present abundant superiority, such as modifiable surface, long-term permeation and metabolism in living body, approved unique properties of BBB penetration, special cells targeting, observed unique therapeutic effects for some diseases, etc. For examples, Rosenkrans *et al.* developed selenium-doped CDs as broad-spectrum antioxidants, and Kim *et al.* indicated the promising application of CDs in preventing α-synucleinopathy for Parkinson's disease 108, 109. On the other hand, comprised with other larger nanomaterials, the smaller CDs (with the size of usually less than 10 nm) exhibit well-organized carbon atoms with a high aspect ratio, large surface area, and high thermal and chemical stabilities, enabling their promising capability for designing different biomaterials and nanomedicines. These CDs-based nanotheranostics have been approved in various works.

**Figure 1:**
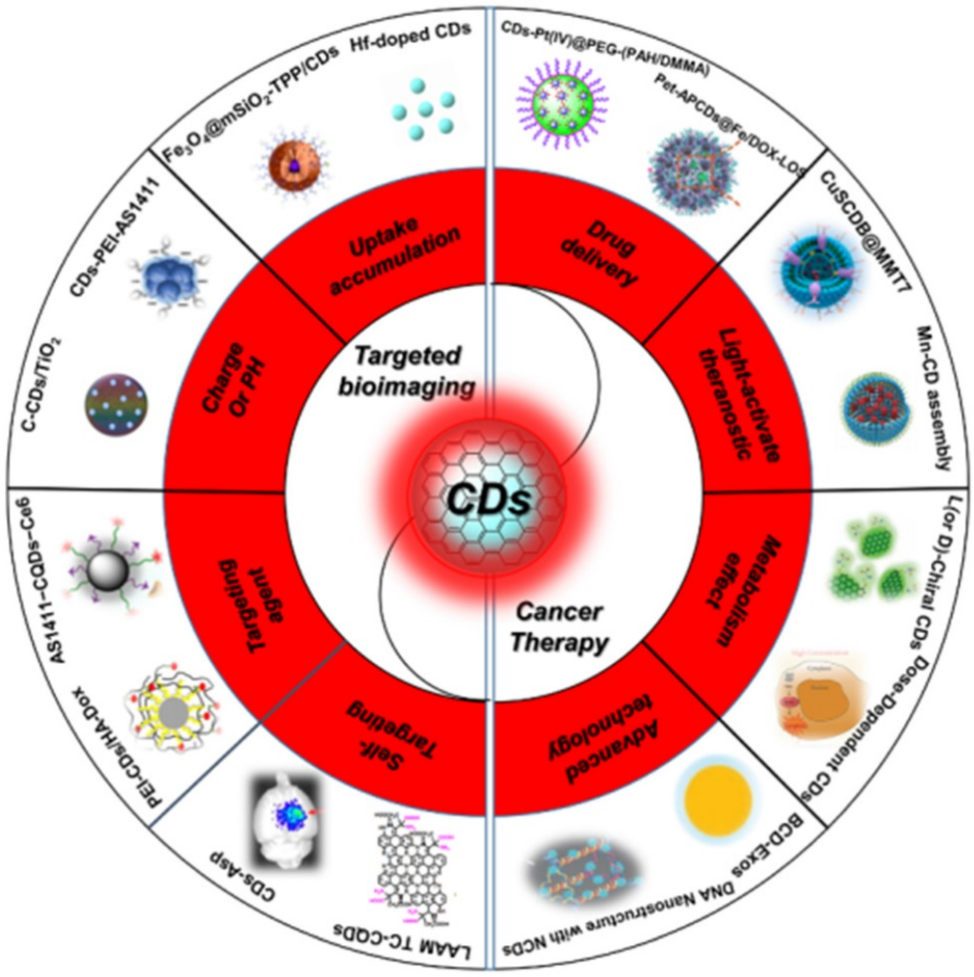
Schematic of the carbon dots in tumor for targeted bioimaging and cancer therapy.

Recently, there have been some reviews about the nanotheranostics focus on bioimaging and cancer therapy, enabling the contrast and precise imaging of cell and organelle or improving the therapeutic effects of several tumor lines 110-115. However, with the development of synthetic technique, the targeted bioimaging and cancer therapy have been expanded to a broader field, such as uptake accumulation, charge or pH, targeting agent, self-targeting, drug delivery, light-activate theranostic, metabolism and precise therapy via different CDs and their nanocomposites (Figure 1 and Table 1) 116-166. Herein, the strategies of CDs-related nanotheranostics to achieve efficient targeted bioimaging and cancer therapy have been reviewed and discussed, and the challenge and prospect in the future development of this emerging field are also discussed and proposed.

## 2. Synthesis and classification of CDs

**Figure 2:**
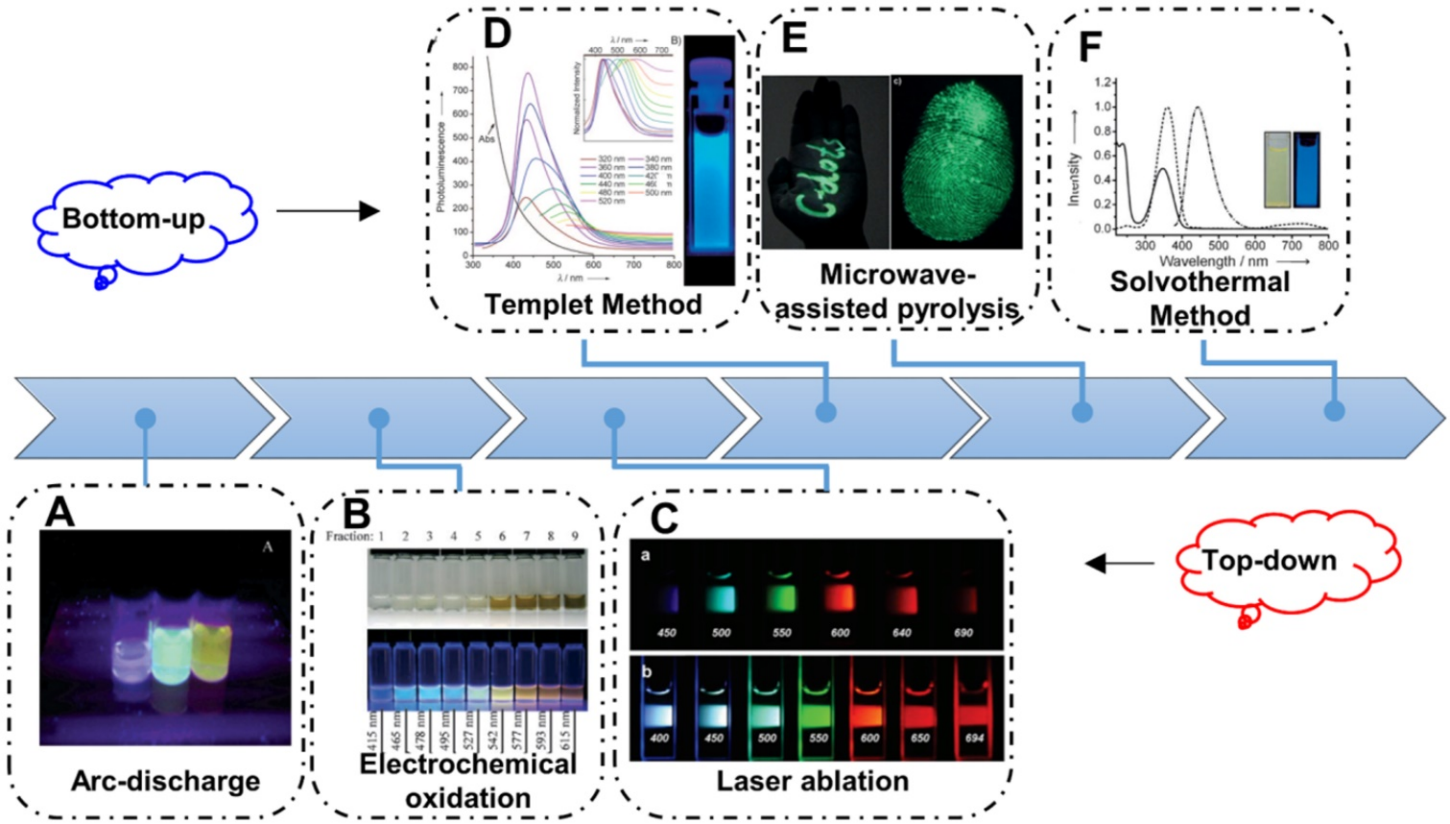
Schematic illustration of the synthetic strategies of CDs. (A) The CDs synthesized by arc-discharge. Adapted with permission from 61, copyright 2004, American Chemical Society. (B) The CDs synthesized by electrochemical oxidation. Adapted with permission from 79, copyright 2007, WILEY‐VCH Verlag GmbH & Co. KGaA, Weinheim. (C) The CDs synthesized by laser ablation. Adapted with permission from 80, copyright 2006, American Chemical Society. (D) The CDs synthesized with templet method. Adapted with permission from 81, copyright 2009 WILEY‐VCH Verlag GmbH & Co. KGaA, Weinheim. (E) The CDs synthesized by microwave-assisted pyrolysis. Adapted with permission from 82, copyright 2012 WILEY‐VCH Verlag GmbH & Co. KGaA, Weinheim. (F) The CDs synthesized with solvothermal method. Adapted with permission from 83, copyright 2013 WILEY‐VCH Verlag GmbH & Co. KGaA, Weinheim.

Since the CDs firstly prepared via electrophoretic purification of single walled carbon nanotubes, various synthetic methods to obtain CDs have been developed (Figure 2). Up to now, the strategies to prepare CDs are always based on the two main routes, naming top-down and bottom-up. Generally, the top-down strategy is considered to obtain nanosize nanoparticles from bulk carbon precursors by broking or exfoliating. Arc-discharge, laser ablation and electrochemical oxidation approaches are the common top-down strategies to achieve broking or exfoliating (Figure 2A-C) 61, 63, 80. In the early stages, the CDs are usually synthesized via the top-down strategy and these CDs always illustrate excitation-dependent emission with low PL QY. Afterwards, with the practical requirement, these top-down strategies are reserved as a paradigm to solve the extensive concern and pioneering studies. By contrast, the bottom-up strategies to prepare CDs are usually considered to obtain nanoparticles by the direct pyrolysis of small molecular. In the early years, researchers developed the template method to prepare CDs with different carriers as template. And the CDs can be obtained in the template through the pyrolysis of carbon-rich precursors, which can accurately control the size and structure of CDs (Figure 2D). With the development of synthetic technology, solvothermal strategy gradually become the most widespread routes to prepare CDs because of its low cost and easy manipulated parameters, such as temperature, time, and pressure of vessel. Among diverse precursors for preparing CDs in solvothermal method, citric acid and its related reactant are the most common ingredients. Since *Zhu et al.* firstly synthesized the fluorescent CDs with the high PL QY of ~80% with citric acid and ethylenediamine, various solvothermal strategies have been employed to achieve fluorescent CDs with high QY through tuning the pyrolysis of small-molecular (Figure 2E) 83. Similarly, microwave-assisted pyrolysis is another fast, low cost and effective bottom-up strategy. In recent years, the microwave radiation has drawn researchers' more attention due to its short reaction times and potential introduced optical properties via special heating principle (Figure 2F). Up to now, fluorescent CDs with ultraviolet to infrared emission have been achieved through bottom-up strategy, implying their great potential for practical applications.

**Figure 3:**
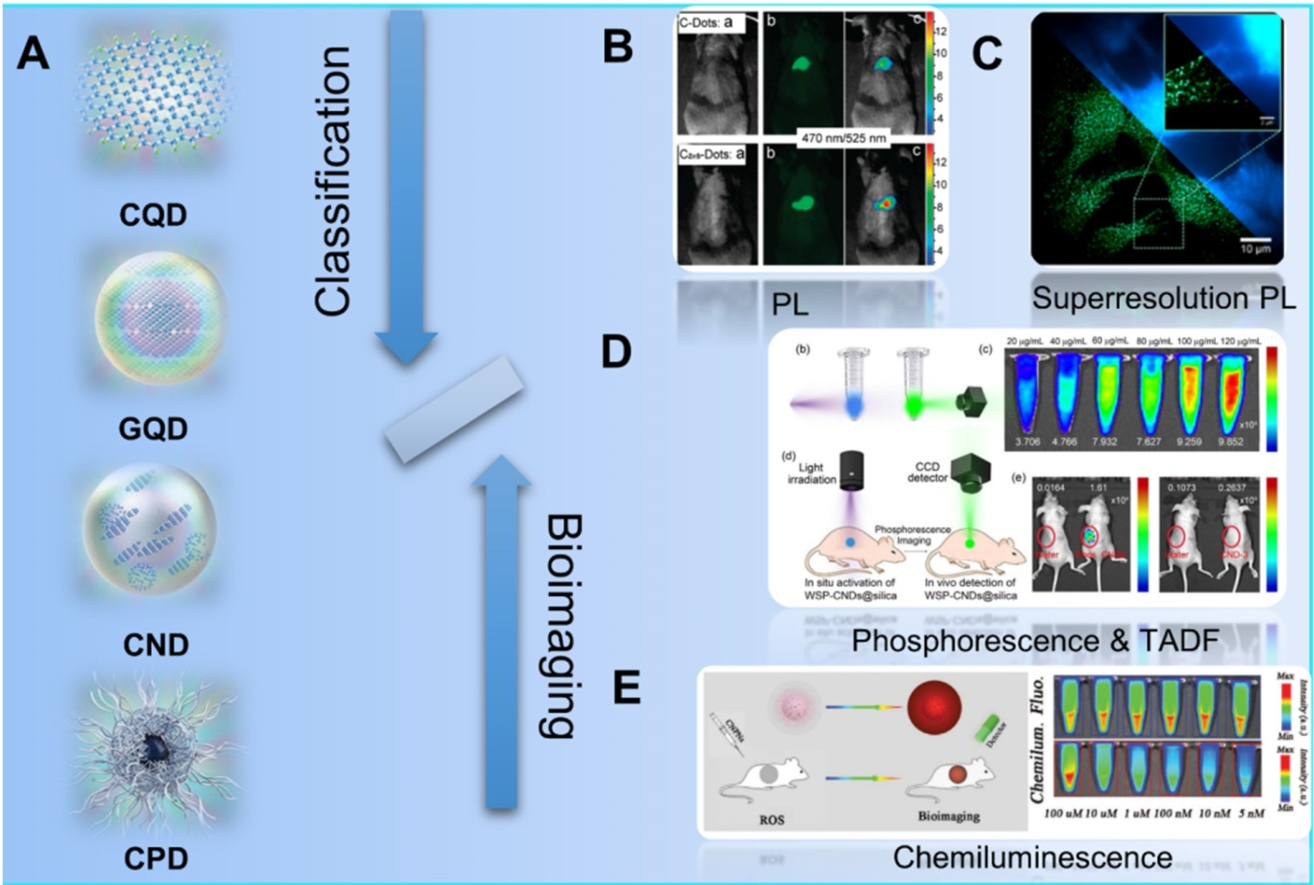
Schematic illustration of the classification of CDs and their different kinds of luminescence models for bioimaging. (A) The classification of CQDs, GQDs, CNDs and CPDs for different CDs. Adapted with permission from 69, copyright 2019 WILEY‐VCH Verlag GmbH & Co. KGaA, Weinheim. (B) Schematic illustration of the PL bioimaging. Adapted with permission from 80, copyright 2009, American Chemical Society. (C) Schematic illustration of the bioimaging via superresolution PL. Adapted with permission from 91, copyright 2018, American Chemical Society. (D) Schematic illustration of the bioimaging by phosphorescence and TADF. Adapted with permission from 143, copyright 2020 Elsevier Ltd. (E) Schematic illustration of the bioimaging with CL. Adapted with permission from 167, copyright 2019 WILEY‐VCH Verlag GmbH & Co. KGaA, Weinheim.

Since the first discover of fluorescent carbon nanoparticles (CNPs) in 2004, diverse kinds of nanoparticles with carbon skeleton have been classified as CDs as shown in the Figure 3. Typically, with the development of synthesis strategies and raw materials, CDs have been defined as one kind of concept of 0 D nanomaterials whose main element is carbon. Recently, Xia *et al.* improved the classification and nomenclature to precisely distinguish different CDs, which are defined as carbon quantum dots (CQDs), graphene quantum dots (GQDs), carbon polymer dots (CPDs), and carbon nanodots (CNDs) via the summary and analysis of structure and properties features for different kinds of CDs (Figure 3A) 92. In these classifications, CQDs are defined as spherical NPs with obvious crystal lattices and chemical groups on the surface, possessing intrinsic state luminescence and quantum confinement effect. GQDs are considered as small graphene fragments consisting of single or few graphene sheets with obvious graphene lattices and chemical groups on the edge or within the interlayer defect, which result in the quantum confinement effect and edge effect. The CPDs are considered as a polymer/carbon hybrid structure comprising of abundant functional groups/polymer chains on the surface and a carbon core. For the CNDs, these NPs possess high carbonization degree with some chemical groups on the surface with no obvious crystal lattices structure and polymer features.

## 3. Targeted Bioimaging

Due to the novel photophysical properties, CDs has been employed as contrast agents for *in vivo* optical imaging (Figure 3B-E) 24, 80, 167-174. In those approaches to optical imaging, the direct PL imaging with light irradiation is the most common strategy. With the down-conversion or multiphoton-excited upconversion fluorescence, the structure of cells or tissues can be reconstructed via the emitted photon of imaging agent (Figure 3B) 83, 90, 93-101. However, the common PL imaging with conventional fluorescence microscopy is limited by the diffraction limit of light by the Abbe criterion. Thereby, two major approaches are employed to overcome the diffraction limit: the patterned illumination-based imaging, including stimulated emission depletion (STED) microscopy and structured illumination microscopy (SIM), and single molecule localization-based imaging, including stochastic optical reconstruction microscopy (STORM) and photoactivated localization microscopy (PALM). For the single molecule localization-based imaging, closely clustered fluorescent particles are resolved by stochastically turning each particle's signal on and off, and then the centroid of on-state particle is mathematically determined in each imaging frame. On the condition, the super-resolution PL image can be reconstructed via the combination of multiple iterations (Figure 3C) 167. In addition to the direct PL imaging, afterglow imaging is considered as another excellent strategy for bioimging. In general, the afterglow imaging through the phosphorescence and TADF can decrease the noise of photoexcited autofluorescence from the background because of the novel delay luminescence (Figure 3D) 13, 69. Similarly, CL is also employed as a contrast optical imaging strategy. Owing to the CL emission as the result of a chemical reaction without photoexcitation, the CL imaging can be employed as special biomolecularr sensor with ultrahigh sensitivity and provide their distinguished bioimging without photoexcited autofluorescence from the background (Figure 3E) 20, 22-27. Thereupon, different strategies have been developed to further achieve targeting bioimaging of cancer cells. In the early stage, researchers found the accumulation effect of CDs in tumors sites and further developed the uptake accumulation targeted imaging. With the development of nanotechnologies, CDs was further developed to achieve tumor targeting imaging with rational design, such as the stimulus-responsive imaging triggered by the novel charge and pH in tumor microenvironment and *in vivo* biomarker imaging with the interaction between CDs and various biomoleculars. More importantly, several researches have observed and approved the special targeting cancer cells marker of CDs in several special tumors, enabling a novel application in self-targeting bioimaging and promising approaches to dignoisis of cancer. Up to now, the CDs-related targeted bioimaging for tumor have been approved for the promising clinical diagnosis for various cancers.

### 3.1. Uptake accumulation

**Figure 4:**
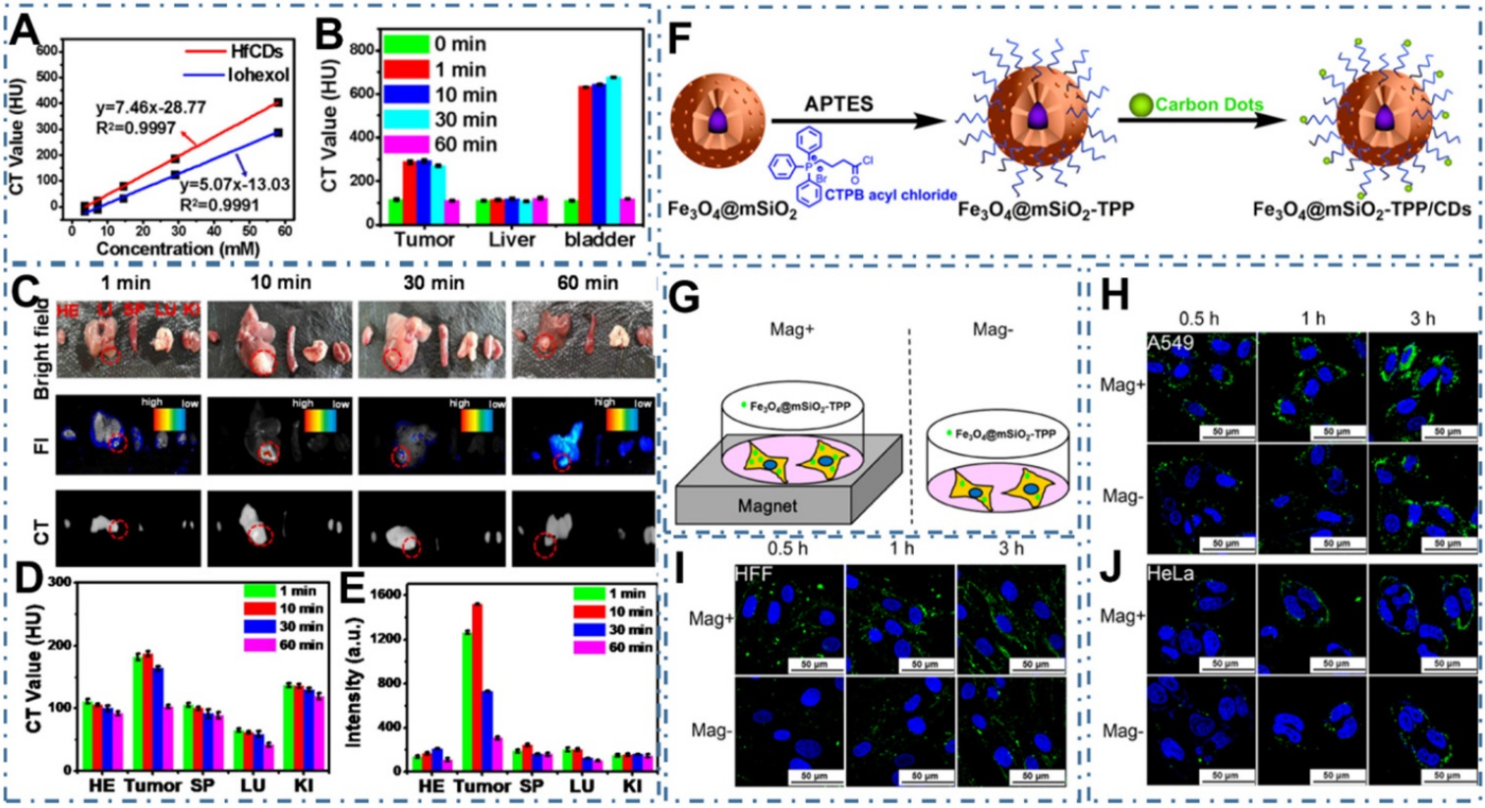
CDs-based targeted bioimaging through uptake accumulation. (A) CT value of Hf-CDs/iohexol aqueous solution at different concentrations. (B) CT values of major organs collected at different times after intravenous injection of Hf-CDs. (C) *Ex vivo* bright field, FI and CT images of major organs collected at different intervals post tail vein injection of Hf-CDs. (D) Quantitative analysis of the CT values. (E) Quantitative analysis of the FI intensity. Adapted with permission from 184, copyright 2020 Elsevier Ltd. (F) Schematic route of the synthesis of Fe_3_O_4_@mSiO_2_-TPP/CDs nanoplatform. (G) Illustration of the cells exposed to the Fe_3_O_4_@mSiO_2_-TPP NPs while positioned or not in a static magnetic field. (H)-(J) CLSM images of the A549 (I), HFF (J), and HeLa (K) cell lines treated with the Fe_3_O_4_@mSiO_2_-TPP NPs for different time under Mag+ or Mag-. Adapted with permission from 185, copyright 2015 American Chemical Society.

As one new class of powerful nanoprobes, CDs have been proved one kind of contrast agents for diverse models of bioimaging since its discover 175, 176. Ideally, the CDs for bioimaging exhibit high PL quantum yield (QY), long-wavelength PL emission, minimum toxicity and renal cleavability, enabling the rational use of inherent CDs in visualizing biological systems both *in vitro* and *in vivo* 177-183. At early stage, CDs exhibit homogeneous permeation and distribution in all cells, endowing similar uptake accumulation for the normal and cancer cells 80, 167. However, several researchers have observed the distinctive accumulation of CDs in tumor tissues due to the enhanced permeability and retention (EPR) effect. For example, Su *et al*. reported the preferential accumulation and efficient renal clearance of CDs at the tumor site 184. In their work, a novel kind of Hafnium-doped CDs (Hf-CDs) exhibited preferential targeted tumor accumulation capability with significant advantages including robust stability, good biocompatibility, excellent water solubility, remarkable computed tomography (CT) contrast performance, enabling the CDs in particular CT/fluorescence imaging for orthotopic liver cancer (Figure 4A-B). In their experiments, researchers found that the Hf-CDs could accumulate at the tumor site for rapid bioimaging (Figure 4C-E), implying a facile and universal method to multimodal imaging. In addition, these researchers further strengthen the distinctive cancer cells uptake accumulation through various approaches. For example, Zhang *et al.* constructed a biocompatible nanoplatform for long time mitochondria-targeting cellular imaging with CDs and further develop the magnetic field-enhanced cellular uptake functionalities to increase the distinctive accumulation 185. With the magnetic mesoporous silica nanoparticles (Fe_3_O_4_@mSiO_2_) via surface modified triphenylphospine (TPP) and conjugated fluorescent CDs (Figure 4F), researchers have proved the novel time-dependent mitochondrial colocalization of the Fe_3_O_4_@mSiO_2_-TPP/CDs nanoplatform in various cell lines such as A549, CHO, HeLa, SH-SY5Y, HFF and HMEC-1. Furthermore, researchers indicated that the cellular uptake efficiency of A549 and HFF cell lines could be enhanced in a short time under a static magnetic field (Figure 4G-J). These distinctive accumulations of CDs in tumors sites provided the basis of targeted uptake and paved the abundant approaches to targeted bioimaging.

### 3.2. Charge or pH

**Figure 5:**
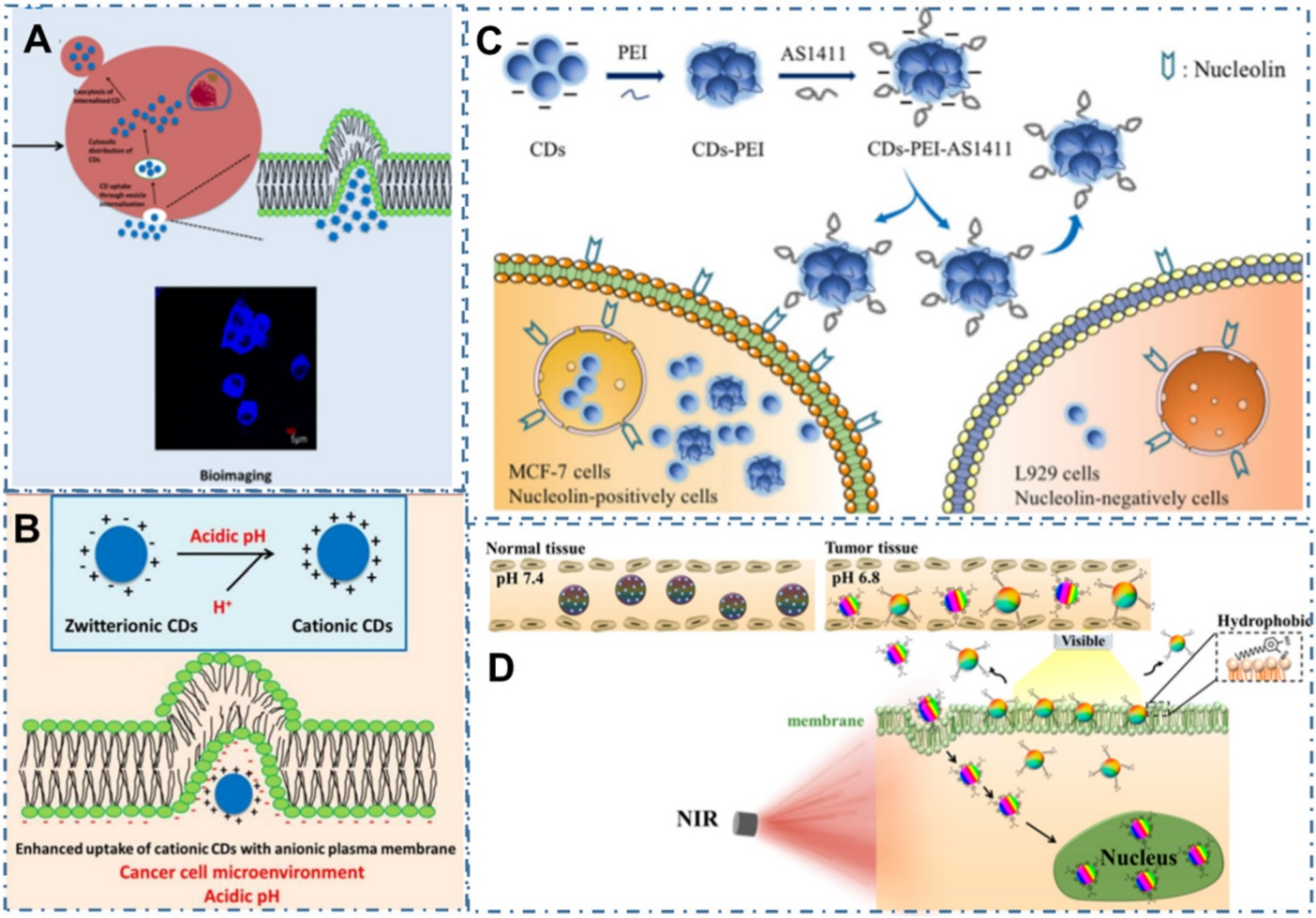
CDs-based targeted bioimaging through charge or pH interaction. (A) Scheme of the synthesis of CDs and its bio-imaging in oral cancer cell lines. (B) Mechanism underlying uptake of zwitterionic CDs. Adapted with permission from 189, copyright 2018 American Chemical Society. (C) Predicted mechanism of the different cellular uptake behaviour of CDs-PEI-AS1411 with the nucleolin-positive MCF-7 cancer cells and nucleolin-negative L929 fibroblast cells. Adapted with permission from 190, copyright 2019 John Wiley & Sons Ltd. (D) Scheme of the design and application of pH-responsive C-CD/TiO_2_ for targeted bioimaging via cellular membrane-nucleus translocation in response to visible-light irradiation. Adapted with permission from 139, copyright 2020 American Chemical Society.

In addition to the direct uptake accumulation, these stimulus-responsive strategies to achieve targeted uptake of CDs based on the distinctive microenvironment in tumor sites, such as charge or the pH, also been explored 186. For example, the zwitterionic CDs can easily bioconjugate with various biomolecules through the interaction between biomolecules and their carboxylic moieties, shed their anionic component and leave a positive charge on their surface when they achieve interaction with cancer cell microenvironment, enhancing their targeted uptake in cancer cells 187-189. On the basis, Sri et al. developed one kind of zwitterionic CDs and indicated their targeted bioimaging for the oral cancer cell lines of FaDu (human pharyngeal carcinoma) and Cal-27 (human tongue carcinoma) (Figure 5A-B) 189. Similarly, Kong et al. developed an efficient detected and targeted nanosystem based on the DNA aptamer AS1411 modified CDs with polyethyleneimine (PEI) as connecting bridge (Figure 5C) 190. In their work, researchers confirmed the higher cellular uptake of CDs-PEI-AS1411 nanosystem in MCF-7 cells compared with that of L929 cells, which revealed the highly selective detection ability of nucleolin-positive cells. In addition to the charge interaction, pH-responsive interaction was indicated another factor to increase the cellular uptake selectivity of free CDs. For example, Phuong et al. developed a selective and sensitive nanotheranostic nanoplatform based on the pH-responsive TiO_2_-integrated cross-linked CDs (C-CD/TiO_2_) for tumor diagnosis by the specific targeted capability of the tumor cell membrane and nuclei (Figure 5D) 139. In their work, researchers designed the zwitterionic-formed CD (Z-CD) which could target the nucleus and the hydrophobic Dopa-decyl (D-CD) which could penetrate the hydrophobic sites of cell membrane. With the boronate ester linkages between the TiO_2_-immobilized D-CD and Z-CD for nuclear targeting, the fluorescence “off” state at physiological pH and the fluorescence “on” state in acidic cancer cells were employed for tumor-selective biosensors through the cleavages of the boronate ester bonds by the disruption of the FRET. In the *in vivo* tumor model, the C-CD/TiO_2_ could efficiently ablate tumors under NIR light irradiation with up-regulation of the pro-apoptotic markers in tumor, illustrating excellent targeted bioimaging and therapy capability.

### 3.3. Targeting biomarker

**Figure 6:**
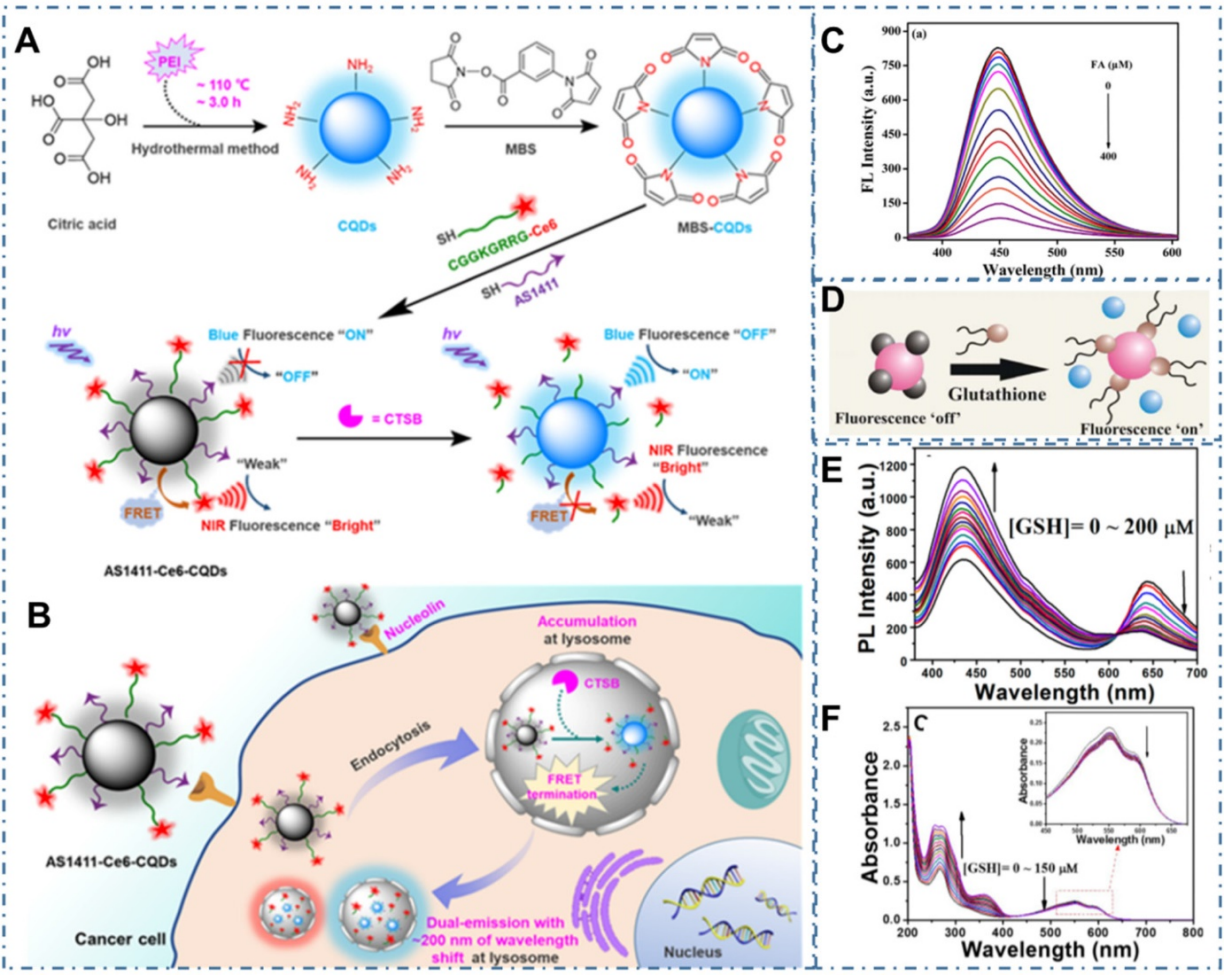
CDs-based targeted bioimaging through targeting biomarker sensor. (A-B) Schematic diagram of the engineering of nucleolin-targeted ratiometric fluorescent nanoprobe AS1411-CQDs-Ce6 for endogenous CTSB imaging with a remarkably large emission wavelength shift in living cancer cells. Adapted with permission from 194, copyright 2020 American Chemical Society. (C) Fluorescence emission spectra of AA-CDs upon gradual addition of FA. Adapted with permission from 195, copyright 2018 Elsevier B.V. (D) Schematic of the glutathione triggered Fluorescence “Turn On” of ACD. Reproduced with permission 196, copyright 2016 American Chemical Society. (E) Fluorescence spectrum of CDs in the presence of various concentrations of GSH. (F) UV-vis spectrum of CDs in the presence of various concentrations of GSH. Adapted with permission from 197, copyright 2020 American Chemical Society.

Beyond the direct targeted imaging of cancer cells, the strategy to *in vivo* image the biomarkers in tumors is another approach to the diagnosis of cancer 191-193. For example, Shen et al. reported one approach to bioimaging Cathepsin B (CTSB), which were one of the most promising biomarkers for numerous malignant tumors, enabling efficient diagnosis of cancers at an early stage 194. In the work, researchers developed one kind of amine-rich CDs and further covalently assembled the nucleolin-targeting recognition nucleic acid aptamer AS1411 and a CTSB-cleavable peptide substrate that tethered with chlorin e6 (Ce6), enabling a cancer-targeting and CTSB stimulus-responsive ratiometric nanoprobe of AS1411-Ce6-CQDs (Figure 6A). With the quenching of blue fluorescence from CDs and NIR fluorescence enhancement from the Ce6 by the efficient fluorescence resonance energy transfer (FRET), the overexpressed CTSB in lysosome could cleave Ce6-Pep and trigger the Ce6 moiety dissociation from AS1411-Ce6-CQDs after selective accumulation in cancer cells, thus leading to the termination of FRET and achieving the ratiometric fluorescence response to CTSB (Figure 6B). Thereby, a vigorous ratiometric fluorescent method could be achieved by integrating a cancer-targeting recognition moiety to report CTSB activity. Meanwhile, Qian et al. prepared one kind of CDs (AA-CDs) and proved that the AA-CDs could selectively recognize folic acid (FA), resulting in fluorescence quenching (Figure 6C) 195. In their work, researchers developed a sensitive approach to FA analysis with a detection limit of 40 nM and further developed one kind of fluorescent nanoprobe (FA-AA-CDs) via the conjugated interaction between FA and AA-CDs, enabling them as a fluorescence turn-on nanoprobes for targeted imaging of cancer cells. In the models of cancer cells with different levels of folate receptors (FRs) expression (Hela, SMMC-7721, and A549 cells), FA-AA-CDs exhibited specially targeted imaging of cancer cells with the accordant relationship between the corresponding PL intensity of these cells and their FRs expression levels. Similarly, Das et al. reported the ACD-GNP nanohybrid which comprised the anionic CDs (ACD) protected gold nanoparticle (GNP) as nanoprobe for imaging glutathione (GSH) 196. These researchers indicated the selective GSH sensing of ACD-GNP nanohybrid based on the GSH-triggered variation between fluorescent indicator ACD and the GNP (Figure 6D). With higher selectivity and sensitivity to GSH than other biothiols, the ACD-GNP hybrid achieved selective imaging of cancer cells. In addition, Li et al. developed one kind of dual-emission CDs and approved their ability of ratiometric GSH sensing in cancer cells 197. With the intrinsic ratiometric fluorescence displacement of as-prepared CDs for GSH sensing (Figure 6E-F), these CDs could be employed as an effective tool to achieve targeted imaging of cancer cells.

**Figure 7:**
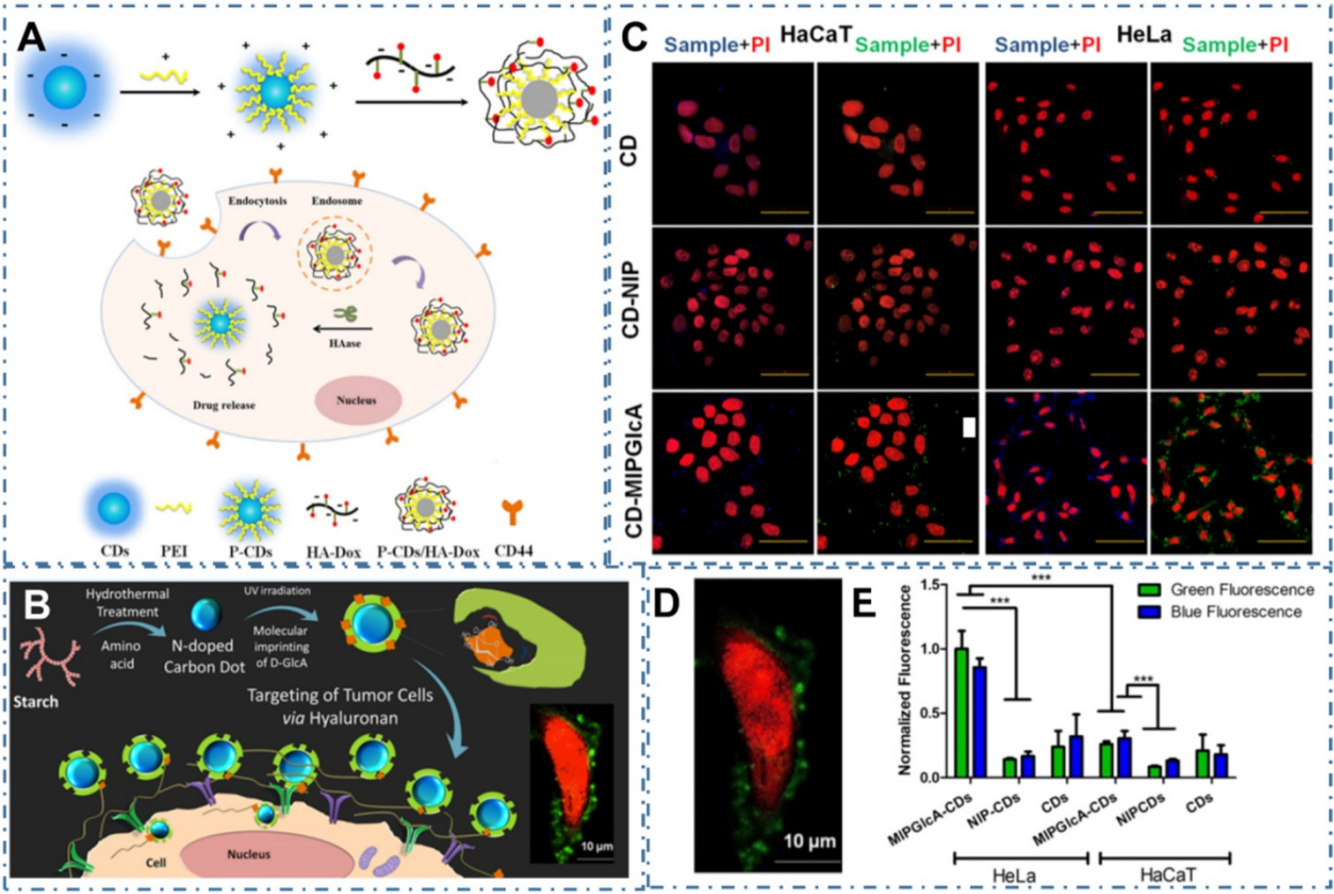
CDs-based targeted bioimaging through selective recognition. (A) Schematic illustration of the formation of PEI-CDs/HA-Dox, and the nanoprobe used for targeted cancer cell imaging and drug delivery. Adapted with permission from 198, copyright 2017 Elsevier B.V. (B) Schematic of molecularly imprinted polymer coated CDs for cancer cell targeting bioimaging, (C) Confocal microscope images of fixed HaCaT and HeLa cells treated with CDs, CD-NIP, and CD-MIPGlcA. (D) Confocal micrographs showing labeling of GlcA on a single HeLa cell by CD-MIPGlcA (green) and nuclear staining with PI (red). (E) Analysis of labeled cells with CD-MIPGlcA, CDNIP, and CD as obtained from Image J by measuring the normalized fluorescence of each single cell area from five different images. Adapted with permission from 199, copyright 2018 American Chemical Society.

As similar as these common strategies to directly sense the biomarker, the approaches to selective recognizing the substance such as antibody or metabolites of cancer cells are also developed. For example, Gao et al. designed one kind of turn-on fluorescent nanoprobes of P-CDs/HA-Dox by electrostatic assembly of PEI-modified CDs (P-CDs) and Hyaluronic acid (HA)-conjugated doxorubicin (Dox) to selectively sense and image hyaluronidase (HAase) (Figure 7A) 198. In their work, the P-CDs/HADox illustrated low PL emission in a physiological environment and were capable to selectively penetrate into cancer cells due to the activation of HAase by utilizing their overexpressed HA to CD44 receptors, resulting in effectively distinguish of cancer cells and sensitive assay of HAase. In addition, Demir et al. coupled one kind of CDs with molecularly imprinted polymers (MIPs) to prepare biocompatible nanoprobes for cancer cells (Figure 7B) 199. In their work, researchers designed a MIP shell round the CDs by the CDs' emission as an internal light source for photopolymerization to specific recognize the glucuronic acid (GlcA), which was a substructure (epitope) of hyaluronan and a biomarker for certain cancers (Figure 7C-D). Their work proved the targeting imaging of hyaluronan and selectively recognized human cervical cancer with the CD-based nanocomposites.

### 3.4. Self-Targeting

**Figure 8:**
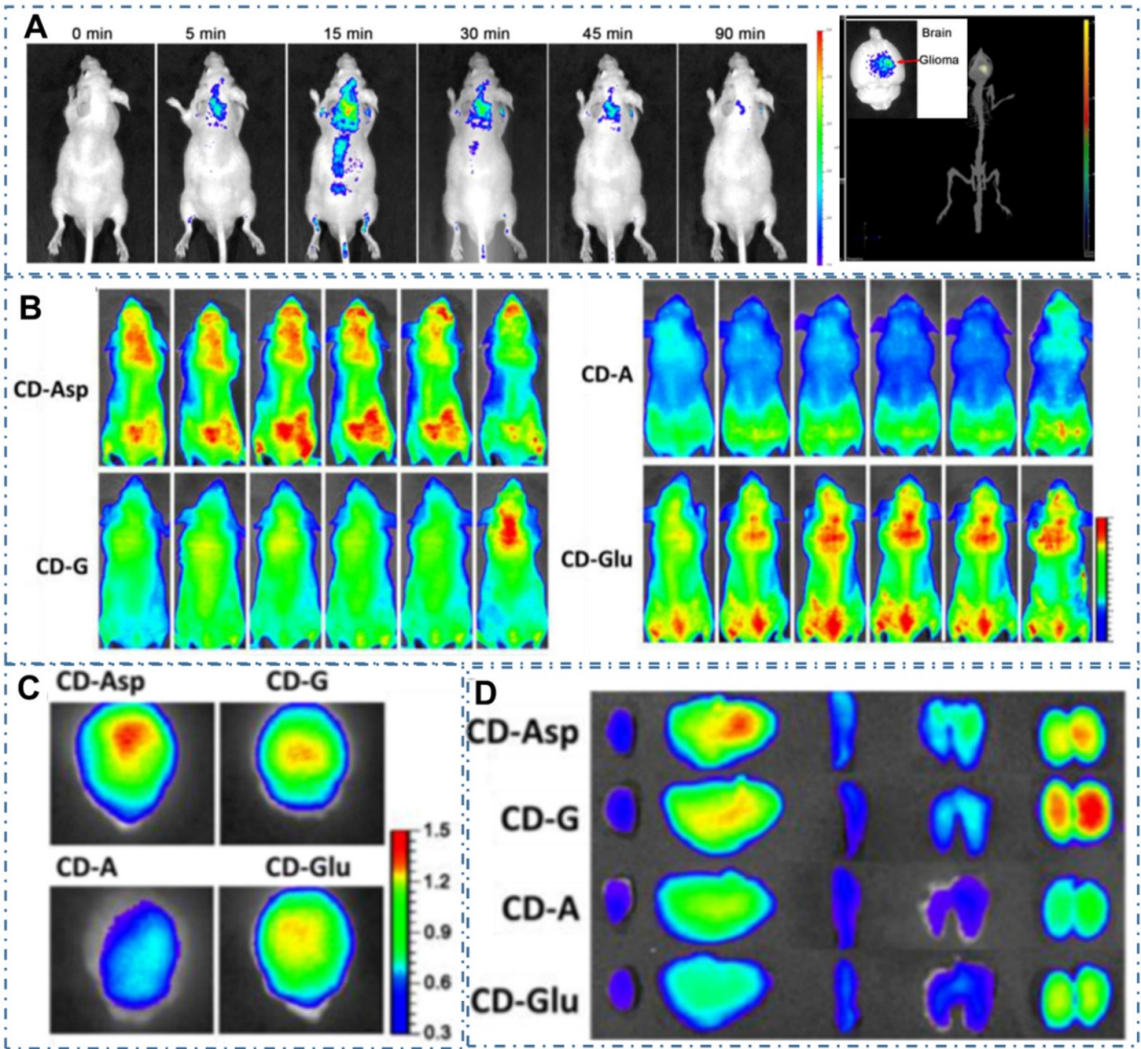
CDs-based targeted bioimaging by self-targeting*.* (A) *In vivo* and *ex vivo* imaging of glioma-bearing mice after tail intravenous injection of CD-Asp. (B) *In vivo* imaging of glioma-bearing mice at different time points after injection with CD-Asp, CD-G, CD-A, and CD-Glu. (C) *Ex vivo* imaging of glioma-bearing brain of brain and glioma. (D) *Ex vivo* imaging after the injection of CD-Asp, CD-G, CD-A, and CD-Glu of heart, liver, spleen, lung, and kidney. Adapted with permission from 200, copyright 2015 American Chemical Society.

**Figure 9:**
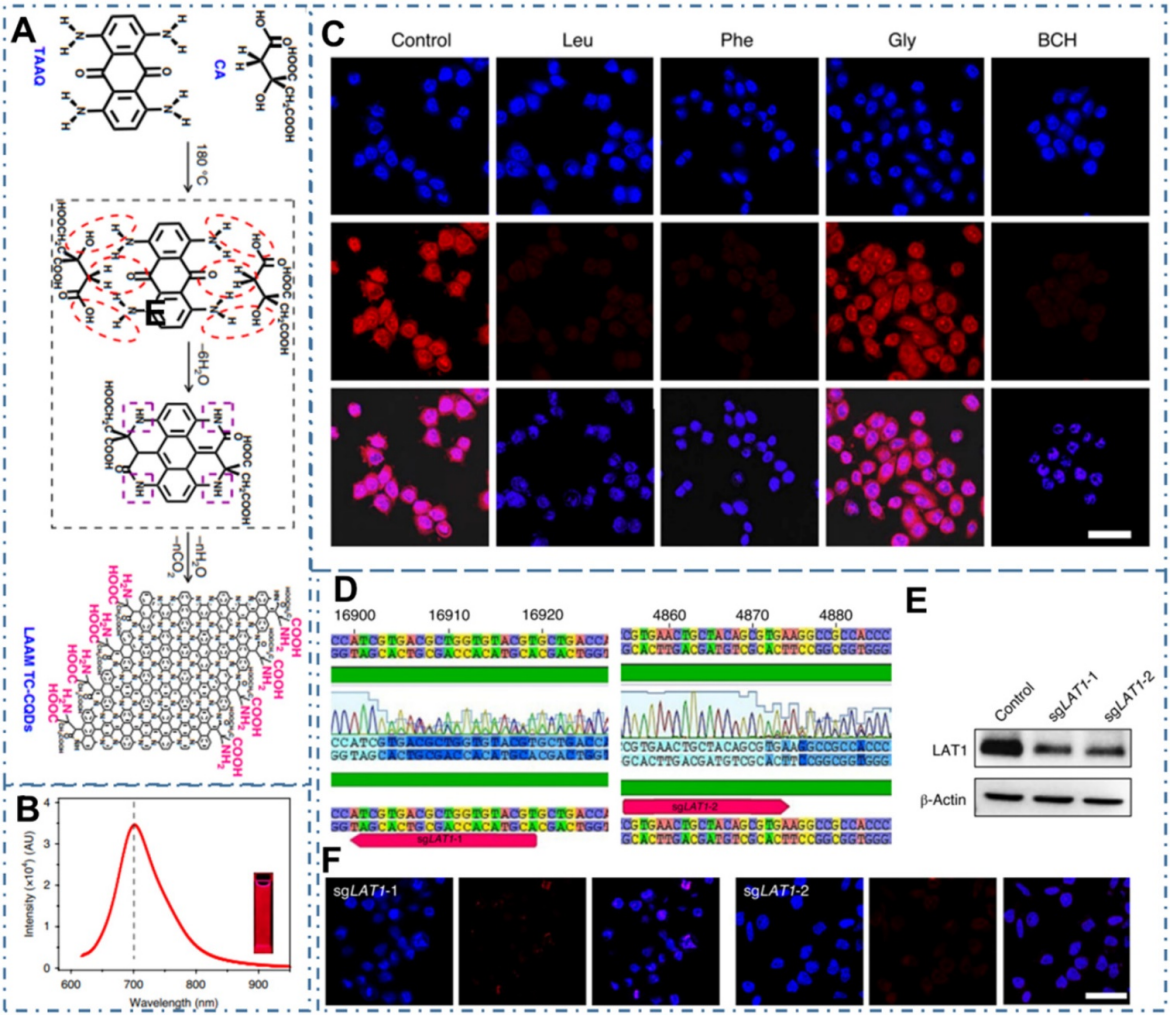
CDs-based targeted bioimaging by self-targeting*.* (A) Schematic and hypothetical steps of LAAM TC-CQD synthesis. (B) Fluorescence emission spectrum with an excitation wavelength of 600 nm. (C) LCSM images of HeLa cells that were pretreated with Leu, Phe, Gly or BCH. (D)-(F) Downregulation of LAT1 expression by CRISPR-Cas9 in HeLa cells reduced the cellular uptake of LAAM TC-CQDs (F). The red arrows in b indicate the sgRNA-targeting sequences. Successful targeting of LAT1 was confirmed using Sanger sequencing (D) and western-blot analysis (E). Adapted with permission from 201, copyright 2020 Springer Nature.

Since the CDs were proved a powerful targeted imaging agent to achieve diagnosis of cancer, these strategies still remain a great challenge to achieve clinic application due to their complex chemical route and potential toxicity. Recently, several kinds of CDs are proved distinctive self-targeting for cancer cells, enabling their promising application in various fields. For example, Zheng *et al.* designed the self-targeted CDs (CD-Asp) with targeting capability to brain cancer glioma through a direct pyrolysis approach with D-glucose and L-aspartic acid 200. The as-prepared CD-Asp with tunable PL emission exhibited selective targeted function toward C6 glioma cells without the requirement of another targeting molecular. In their work, the *in vivo* bioimaging with CD-Asp as fluorescent imaging agent exhibited high-contrast targeted distribution and the higher fluorescent intensity obtained in the glioma site than that in normal brain, enabling the capability of CD-Asp for free penetration in the blood-brain barrier and precise self-targeting of glioma tissue (Figure 8A-D). Generally, there are two major mechanisms by which various agents can cross the BBB: transporter- and receptor-mediated transports. Glucose transporter (GLUT-1) is a brain-tumor-targeting property through facilitative glucose metabolism by the glucose transporters. ASCT2 is an important L-isomer-selective transporter across the BBB through L-glutamine (L-Glu) and L-asparagine (L-Asp) as high-affinity substrates. In the work, researchers observed that the CDs synthesized with glucose, L-Asp, and/or L-Glu and containing the reactant functional groups (glucose, L-Asp, L-Glu) could help them cross the BBB through the GLUT-1 and ASCT2 transporters. Therefore, RGD, a tripeptide composed of L-arginine, glycine, and L-aspartic acid, was a common glioma-targeting agent that binding to RVβ3 integrin on immature endothelial cells. Thereupon, researchers deduced that the targeting function of CD-Asp originated from the formation of RGD-like functional groups on the CDs' edge, which were prepared and derived from D-glucose and L-Asp. Similarly, Li *et al.* developed a series of self-targeted CDs (LAAM TC-CQDs) which functionalized with multiple paired α-carboxyl and amino groups and indicated their selective accumulation in tumor 201. In their work, the LAAM TC-CQDs showed bright PL emission with a *NIR* PL emission peak around 700 nm (Figure 9A-B). With the treatment of the excess of Leu, Phe or Gly before adding LAAM TC-CQDs for HeLa cells, researchers found that the cell uptake of LAAM TC-CQDs was significantly inhibited by Leu and Phe but not by Gly (Figure 9C). On the basis of different experiments, researchers indicated that the LAAM TC-CQDs could penetrate into cancer cells via the interaction with LAT1 and approved this hypothetical mechanism with six lines. First, the pretreatment with the LAT1 inhibitor BCH reduced the uptake of LAAM TC-CQDs (Figure 9D). Second, LAT1 knockout reduced the uptake of LAAM TC-CQDs (Figure 9E-F). Third, overexpressing LAT1 of HeLa cells via lentiviral transduction increased the cellular uptake of LAAM TC-CQDs. Fourth, the expression level of LAT1 correlated with the amount of LAAM TC-CQDs in diverse cell lines and the level of LAT1 expression in cancer cells was higher than that in normal cells. Fifth, the overexpression of LAT1 in tumors increased the uptake LAAM TC-CQDs *in vivo*. Sixth, the pretreatment with Leu decreased the accumulation of LAAM TC-CQDs in tumors. With the functionalized CDs which could load aromatic drugs through π-π stacking interactions, researchers further achieved the *NIR* fluorescence and photoacoustic imaging for various tumors and the targeted drug delivery for the chemotherapeutics to the tumors.

With these excellent performances in bioimaging, CDs exhibit great potentials for the diagnosis of cancer in clinic 173, 174. In practical, Wang *et al.* have employed CDs in the clinical application and demonstrated their excellent performance for guiding the precision surgery of papillary thyroid carcinoma 173. In their reports, researchers evaluated the application of CDs as lymphatic tracer in total thyroidectomy and bilateral Central District's thyroidectomy for papillary thyroid carcinoma. The related results confirmed the CDs can distinguish the thyroid tissue from the surrounding lymphoid adipose tissue and clearly mark the Central District lymph nodes, resulting in the decrease for the risk of parathyroid gland injury during the thyroid cancer. Therefore, the relevant research has confirmed the capability of CDs for targeted cancer bioimaging and approved their great potentials of CDs for clinic applications in future.

## 4. Targeted Cancer Therapy

As CDs accumulate specifically in tumors, they are ideal for further use in targeted cancer therapy 17, 202-235. Meanwhile, with CDs' excellent performance in various bioimaging techniques, it is possible to develop nanotheranostic strategies that can reveal simultaneous diagnosis and treatment of various cancers. At an early stage, CDs are demonstrated to be excellent nanocarriers to reunite with different drugs to enhance targeted therapeutic effect. As nanomaterials develop, CDs have also been used as a new class of nanocarriers for creating nanocomposites with a series of functional nanoparticles such as Au NPs, Fe_3_O_4_ NPs, and some inorganic quantum dots, endowing them with additional characteristics, such as oxidative stress amplifier, magnetic functions and radioactivity, for advanced therapeutic applications. In addition, the special photophysical properties, such as strong absorbance of *NIR* light and/or excellent photothermal/photodynamic character, endow various CDs for light-active nanotheranostic of cancer. Compared with the traditional chemotherapy, the light-active nanotheranostic have received more and more attention due to its noninvasive and stimuli-responsive features and the promising characters to over drug-fast. In addition, several kinds of CDs have been observed distinctive cytotoxicity with novel metabolic pathway in cell growth and death, enabling their direct application for cancer therapy as nanomedicines. Finally, with the development of nanotechnologies, CDs also play important role in various advanced technologies and exhibit excellent applications in several kinds of cancer to overcome the limitation of common medicine.

### 4.1. Drug delivery

**Figure 10:**
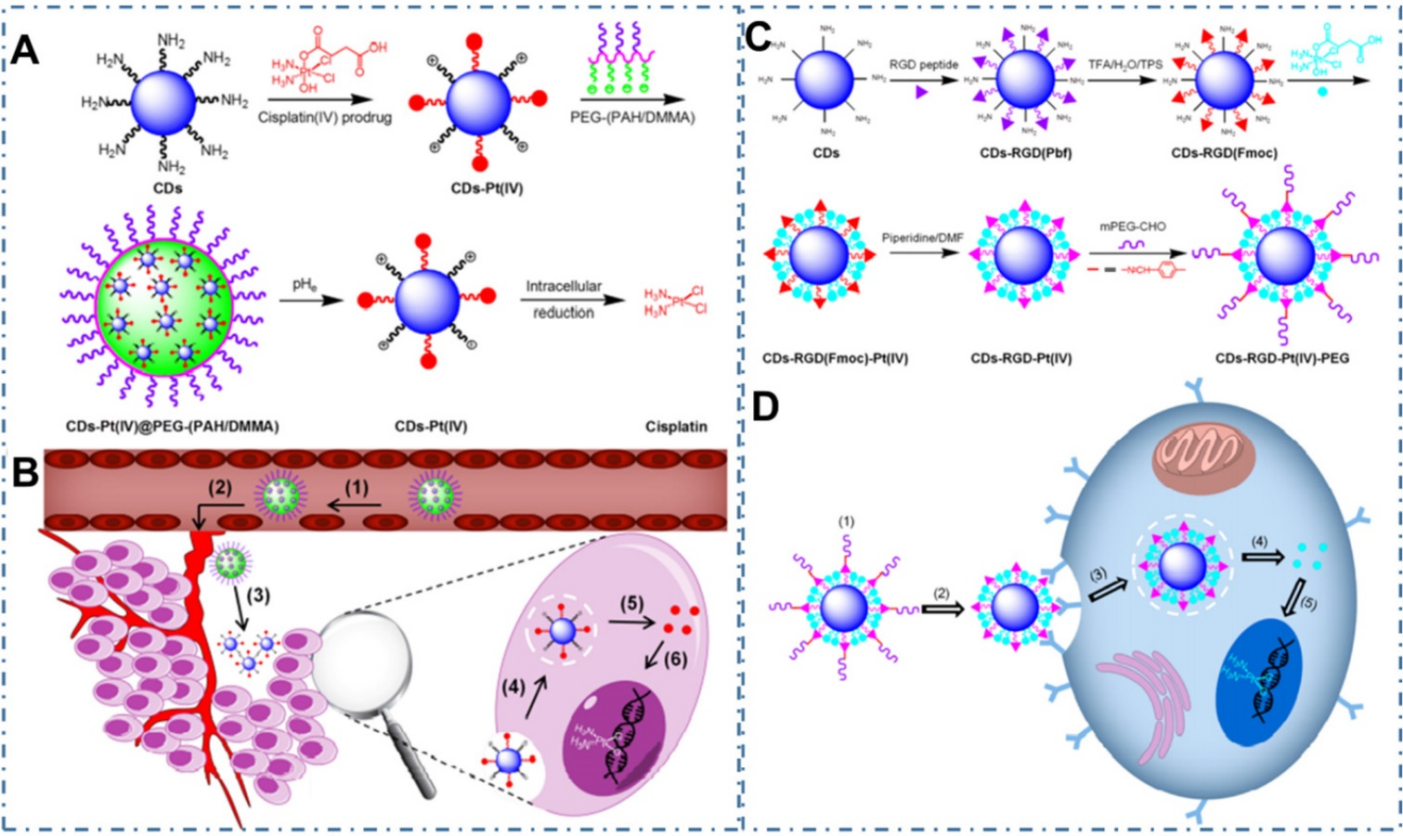
CDs-based cancer therapy via drug delivery. (A) Schematic illustration for the preparation of charge-convertible CDs-based drug nanocarrier CDs-Pt(IV)@PEG-(PAH/DMMA). (B) Schematic illustration for the drug delivery process of CDs-Pt(IV)@PEG-(PAH/DMMA). Adapted with permission1 from 207, copyright 2016, American Chemical Society. (C) Schematic illustration for the preparation of CD-based drug nanocarrier CDs-RGD-Pt(IV)-PEG with tumor-triggered targeting property. (D) Schematic illustration of the drug delivery process using CDs-RGD-Pt(IV)-PEG. Adapted with permission from 208, copyright 2016 American Chemical Society.

**Figure 11:**
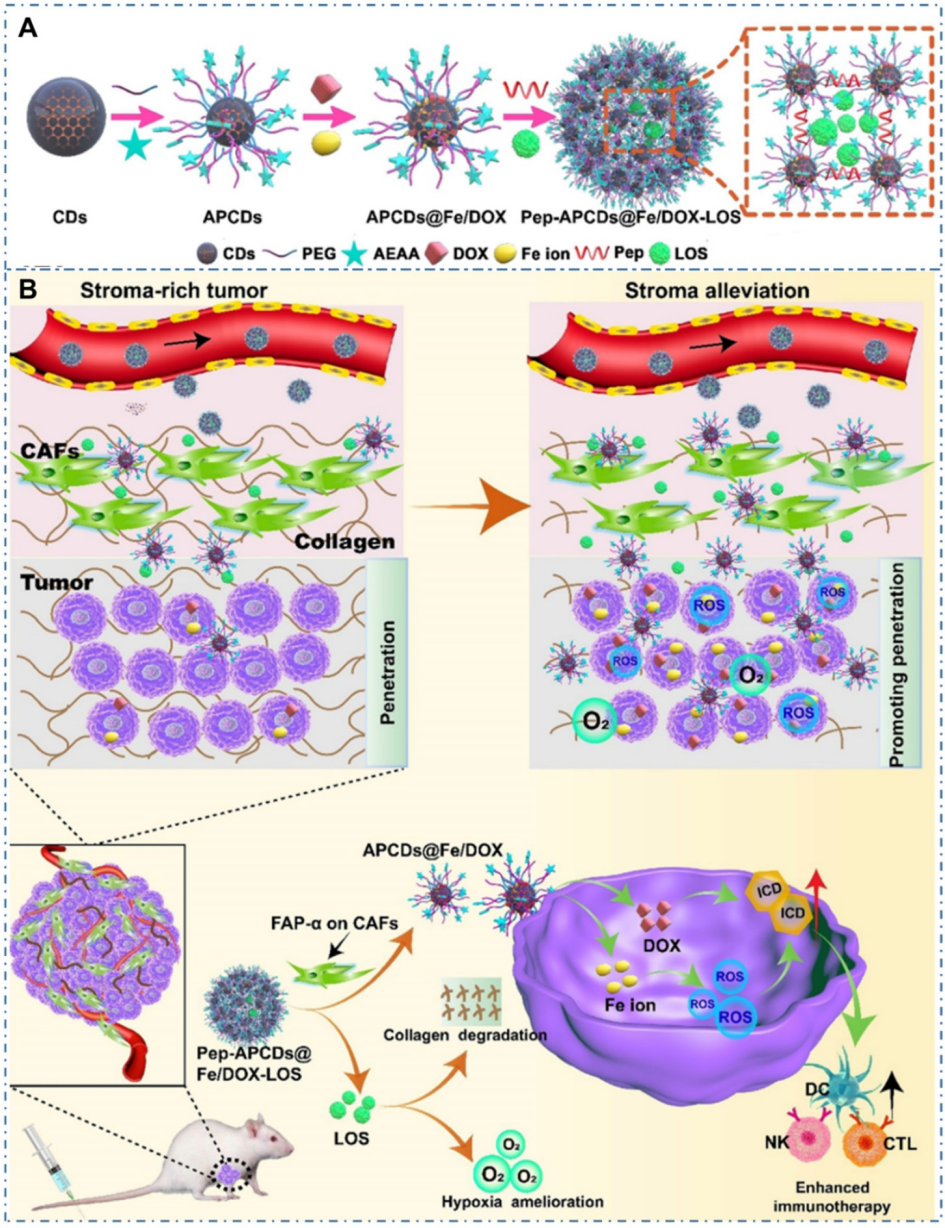
CDs-based cancer therapy via drug delivery. (A) Schematic illustration for the preparation of drugs-loaded nanoassemblies of Pep-APCDs@Fe/DOX-LOS. (B) The transformation and enhanced antitumor immunity mechanism of Pep-APCDs@Fe/DOX-LOS. Adapted with permission from 219, copyright 2020 Wiley-VCH GmbH.

Chemotherapy is the most common strategy for diverse cancers. Nevertheless, the limitation such as potential drug resistance and ineffectiveness against metastatic disease and lack of an effective targeting of the classical drug for cancer therapy seriously restrict their therapeutic effect in living body. Thereupon, different nanotechnologies are developed to achieve enhanced targeted therapy for tumors. In the field, CDs also are employed as nanocarries to achieve precise drug delivery through their novel surface structures to load drugs or distinctive targeted release capability triggered by tumor microenvironment. Meanwhile, besides both excellent optical imaging quality and drug delivery efficiency, these CDs used for nanocarriers are demonstrated few or nontoxic effects with *in vitro* and *in vivo* studies. And these CDs-based nanomedicines illustrate excellent therapeutic effect (Table 2). The maximal drug loading capability of the CDs approach 96% with no noticeable cell growth inhibition, morphological damage or inflammatory injury. For example, Feng *et al.* designed a drug nanocarriers with the responsivity of tumor extracellular microenvironment based on the cisplatin(IV) prodrug-loaded CDs (CDs-Pt(IV)@PEG-(PAH/DMMA)) for bioimaging-guided drug delivery (Figure 10A-B) 207. In their work, the anionic polymer of dimethylmaleic acid (PEG-(PAH/DMMA)) on the CDs-Pt(IV)@PEG-(PAH/DMMA) could pass through charge conversion to the cationic polymer in the tumor extracellular microenvironment (pH∼6.8), enabling the strong electrostatic repulsion and release of positive CDs-Pt(IV). In addition, the nanocarrier with positive charge displayed high affinity to cancer cell membrane with negative charge, which resulted in the increase of internalization and effective activation of cisplatin (IV) prodrug. The *in vitro* experiments indicated that this promising exhibited the better therapeutic efficiency of the charge-convertible nanocarriers under tumor extracellular microenvironment than the normal physiological condition and the noncharge-convertible nanocarriers. The *in vivo* experiments further approved the high tumor-inhibition efficacy and low side effects of these charge-convertible CDs, implying their capability as promising drug nanocarriers in practical clinical application. On the condition, Feng *et al.* further developed the pH/redox dual-responsive CDs (CDsRGD-Pt(IV)-PEG) for tumor extracellular microenvironment responsive targeted bioimaging and enhanced anticancer drug delivery (Figure 10C-D) 208. In their work, the CDsRGD-Pt(IV)-PEG were constructed with fluorescent CDs as drug nanocarriers, cisplatin(IV) as prodrug, and RGD peptide as active targeting ligand by the cover of monomethoxypolyethylene glycol (mPEG) via the pH responsive benzoic-imine bond in tumor extracellular microenvironment (6.5∼6.8). The drug nanocarriers could be tracked by the fluorescence of CDs and illustrated effective uptake in cancer cells via RGD-integrin αvβ3 (ligand-receptor) interaction after the hydrolysis of benzoic-imine bond in the tumor extracellular microenvironment to release the inner RGD peptide. Although these nanocarriers were promising to overcome the biological barriers in the process of drug delivery, the fibrosis in tumor sites could cause hypoxia, immunosuppression and limited immunocytes infiltration, thus reducing the antitumor curative effect of various nanosystems. Thereupon, Hou *et al.* designed one kind of honeycomb-like nanoassemblies of CDs with cancer associated fibroblasts (CAFs) responsivity to spatially program the delivery of therapeutics for enhanced antitumor efficiency (Figure 11A-B) 219. In their work, the doxorubicin (DOX) and immunotherapeutic enhancer (Feions) were attached on the CDs, and the tumor microenvironment modifier (losartan, LOS) was encapsulated within the mesopores. Their experiments indicated the drug-loaded nanoassemblies could be disunited to release LOS to mitigate stroma and hypoxia with the responsivity to CAFs, and the individual CDs with DOX and Fe ion could efficiently penetrate into tumor sites to enhance immune responses. The *in vitro* and *in vivo* experiments indicated that the nanoassemblies exhibited effective T-cells infiltration, tumor growth inhibition and lung metastasis prevention. These works provided the new therapeutic nanoplatforms for diverse tumors.

**Figure 12:**
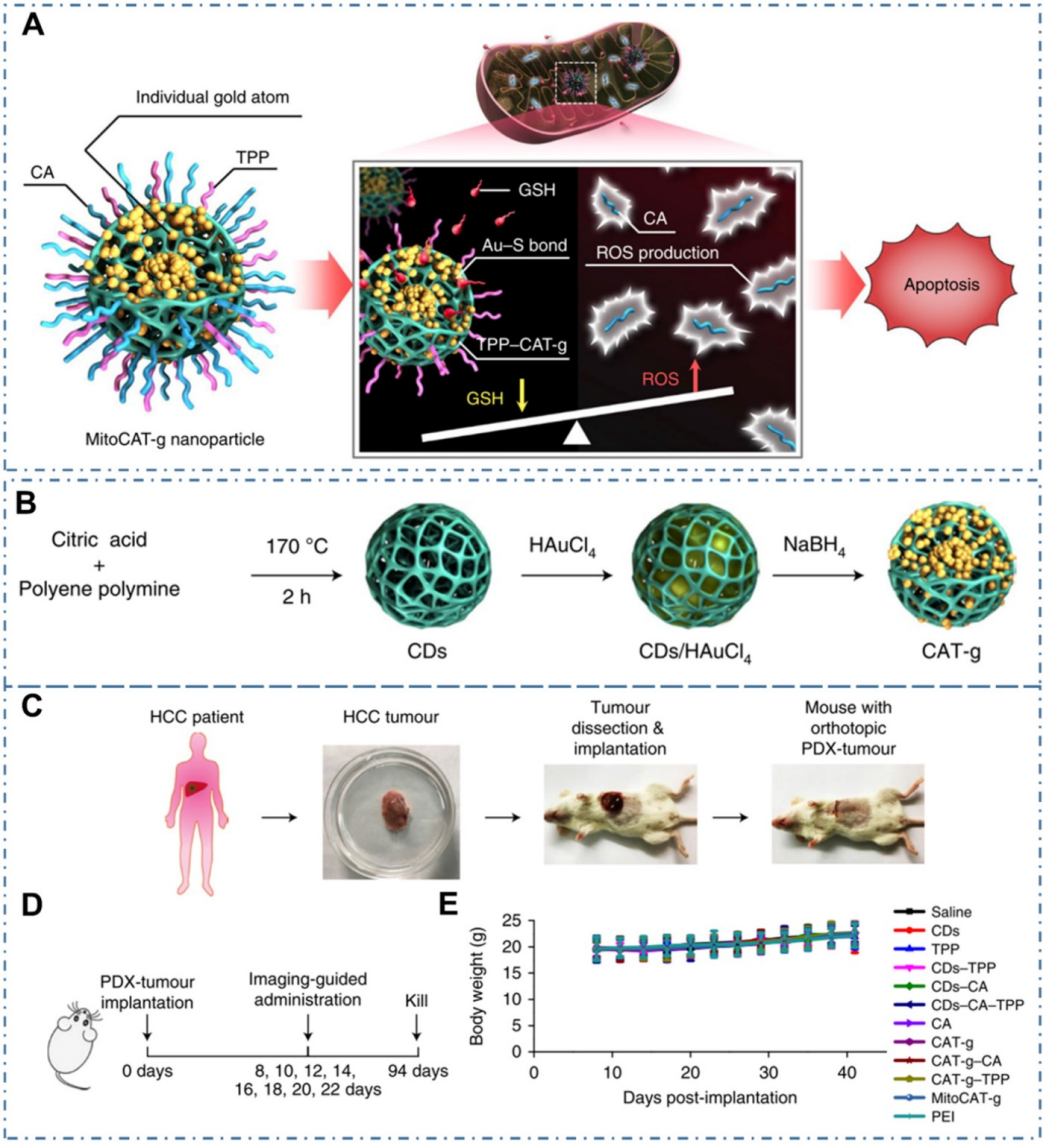
CDs-based cancer therapy via drug delivery. (A) Design and mechanism of MitoCAT-g to amplify oxidative stress in mitochondria and cause apoptotic cell death. (B) Schematic illustration of the synthesis process of CAT-g. (C) Schematic illustration of the establishment of the orthotopic PDX tumour model in NOD-SCID mice. (D) Scheme of the treatment schedule. (E) Effect of the different formulations on animal body weight. Adapted with permission from 221, copyright 2019 Springer Nature.

Similarly, CDs may be used as nanocomposites, composited with other nanomaterials, so as to provide additional characteristics. For example, Gong *et al.* reported a mitochondrial oxidative stress amplifier of MitoCAT-g with CDs and indicated that the MitoCAT-g particles could selectively target mitochondria and deplete mitochondrial GSH with atomic economy, thus amplifying the ROS damage and resulting in apoptosis of cancer cells (Figure 12A) 221. In their work, the MitoCAT-g was designed by CDs-supported atomically dispersed gold (CAT-g) with further surface modifications of triphenylphosphine (TPP) and cinnamaldehyde (CA) (Figure 12B). With the *in vivo* experiments, the capability and mechanism of MitoCAT-g to suppress tumor growth in a subcutaneous tumor model were proposed (Figure 12C-D). With the *in vivo* tumor-bearing mice models, all the group showed a significant change in body weight (Figure 12E) and the MitoCAT-g group exhibited a significant inhibited tumor growth compared with the saline, CDs, TPP and CDs-TPP treated groups. Meanwhile, the MitoCAT-d-treated group showed 87.5% survival and the antitumor effect was reversed in HepG-2 (SOD2-mito-CAT) tumors. These results confirmed the effectiveness of CDs-based nanocomposites for antitumour application and represent a promising medicine for clinical anticancer applications.

With these excellent optical properties and low toxicity (Table 2), CDs exhibit promising potential application in the nanotheranostics. Though most of CDs exhibit low toxicity, there are still widespread concerns about the risk of CDs for therapy applications. On the one hand, there have been various animal studies revealing that other carbon family nanomaterials like CNTs, can be internalized by macrophages and induce inflammation and injury in the respiratory system. So far, reports of CDs for therapy applications have employed in different tissues. There still are no reports about the inflammatory response or neoplastic lesions known about whether CDs exposure may eventually affect cancer progression via possible systemic effects on non-adjacent organs. On the other hand, there have been several reports about the novel cytotoxicity in some kinds of CDs, such as their ROS-generation toxicity, dose-dependent toxicity, etc. Thereby, the risk of CDs for clinic therapy applications still needs more concerns and studies.

### 4.2. Light-activated theranostic

**Figure 13:**
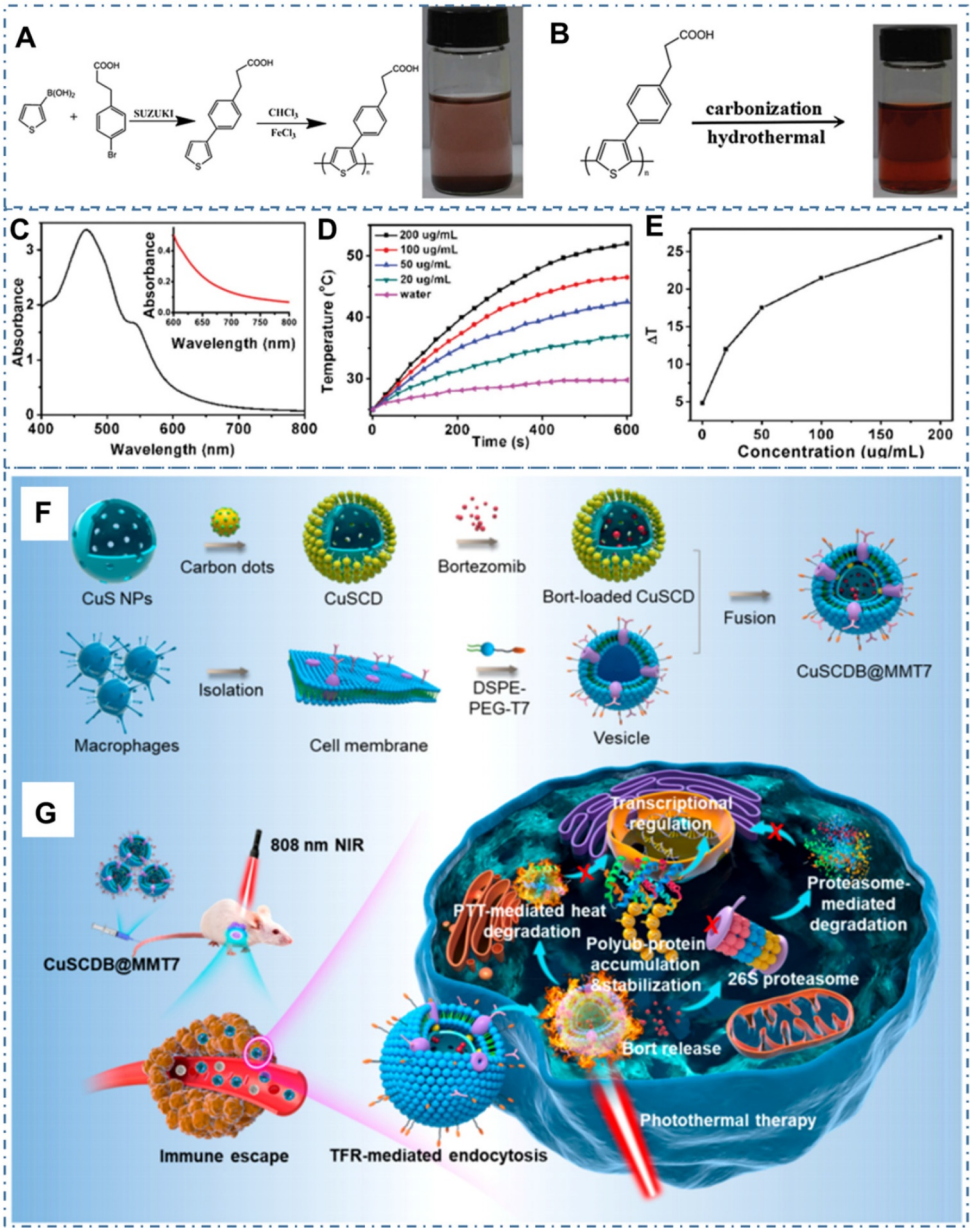
CDs-based cancer therapy by PTT. (A) Synthetic route of PPA. (B) Synthetic route of CDs. (C) The absorption of water-dispersible CDs. (D) Temperature elevation of pure water and the aqueous dispersion of CDs with different concentrations under laser irradiation. (E) Plot of temperature change over a period of 600 s versus the aqueous dispersion of CDs with different concentrations. Adapted with permission from 116, copyright 2015 WILEY-VCH Verlag GmbH & Co. KGaA, Weinheim. (F) Schematic illustration of the generation of proteasome inhibitor-encapsulated CuS/carbon dots nanocomposites (CuSCDB@MMT7). (G) Schematic illustration of the application of CuSCDB@MMT7 for enhanced PTT via heat-stabilization of various substrates in the ubiquitin-dependent proteasomal degradation pathway. Adapted with permission from 129, copyright 2020, American Chemical Society.

Beyond traditional drug delivery, light-activated nanotheranostics are considered as the most promising cancer therapy strategy in clinic 53, 118, 222, 223. As the important modalities of light-triggered treatment such as photothermal therapy (PTT) and photodynamic therapy (PDT), utilizing photoabsorbing molecules or nanoparticles to convert absorbed light energy to heat or generate reactive oxygen species (ROS), consequently achieving local hyperthermia for efficient cancer therapy. In the last ten years, the CDs and their composites are skillfully designed to satisfy the requirement of practical treatment, such high NIR absorbance, excellent biocompatibility and low nephrotoxicity. In the process, the intrinsic characters of CDs or the obtained properties of CDs-based nanocomposites combined with other assembled materials are gradually improved and perfected with various technologies. In these strategies, PTT is one kind of promising therapeutic strategy for diverse cancer while it is still a critical challenge in the rational design of photothermal agent with effective photothermal conversation for the therapeutic outcome. In general, the main differences of PTT properties from photothermal agents are based on the following three parameters: the photothermal conversion efficiency (PCT), absorption cross section and selectivity of light absorption. In these concepts, the PCT is the ratio of conversed thermal power to excited light power, which directly decides the treatment performance of PTT materials under same condition. The absorption cross section is based on the particles' geometric cross section and light absorption efficiency, which determines the absorption capability of materials under the same powerful light. The selectivity of light absorption refers to the light absorption efficiency for different lights. Due to the scatter of tissues, the therapeutic window in the near infrared region from 650 to 1000 nm has a high tissue penetration depth in comparison to the visible light, which is benefited in the application of light-active treatment. The photothermal properties of CDs with different sizes have been summarized in Table 1. The maximum PCT of CDs can reach 78% under the excitation of 808 nm near-infrared light. And the effective light absorption range has been extended to NIR-Ⅱ region with the light absorption greater than 1000 nm. For the CDs with photothermal capability, researchers have designed and tuned the intrinsic photothermal conversation property by rational raw materials and chemical route for the preparation. For example, Ge et al. designed the CDs with intrinsic photothermal conversation by choosing precursor molecules of polythiophene derivatives (PT2) and then further carbonized the polymer of polythiophene phenylpropionic acid (PPA) (Figure 13A-B) 116. The as-prepared CDs exhibited a broad absorption band from the visible to NIR region with a red emission (Figure 13C) and efficient photothermal conversion efficiency (Figure 13D-E). With the novel ability, researchers employed the CDs as simultaneous fluorescence, PA and thermal theranostics for cancer diagnosis and therapy in living mice and achieved excellent performance. In addition to the intrinsic PTT, CDs also are employed as nanoplatforms to assemble with other materials to enhance photothermal conversation. For example, Yu et al. rationally designed one kind of hollow-structured CuS nanoparticles composited with CDs to form CuSCDs and proved their high photothermal conversion efficiency with excellent biocompatibility and low toxicity (Figure 13F) 129. With the coating of a macrophage membrane hybridized with T7 peptide on the surface of the proteasome inhibitor loaded CuSCD, the obtained CuSCDB@MMT7 illustrated selective specificity to cancer cells with the characteristics of immunity escaping and increased transferrin receptor-mediated endocytosis (Figure 13G). Meanwhile, the CuSCDB@MMT7-triggered PTT showed the accumulation of the polyubiquitinated tumor suppressor protein that was heat stabilized under NIR induced hyperthermia, facilitating augmented tumor cell apoptosis and the attenuated metastasis.

**Figure 14:**
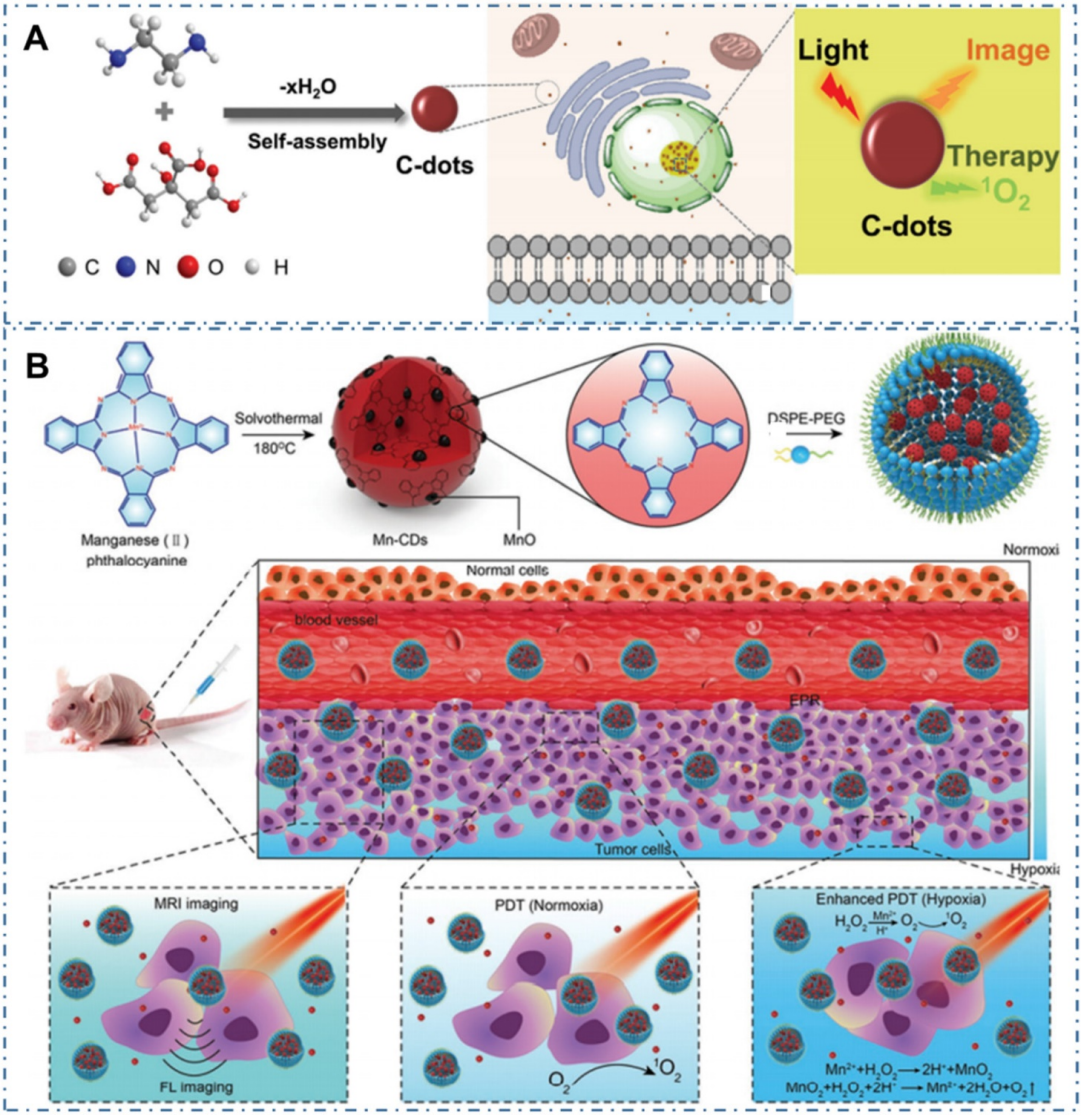
CDs-based cancer therapy by PDT. (A) Schematics of synthetic procedure of CDs and corresponding nucleolus-targeted photodynamic anticancer therapy. Adapted with permission from 224, copyright 2020 WILEY-VCH Verlag GmbH & Co. KGaA, Weinheim. (B) Schematic illustration of the Mn-CD assembly as an acidic H_2_O_2_-driven oxygenerator to enhance the anticancer efficiency of PDT in a solid tumor. Adapted with permission from 225, copyright 2018 American Chemical Society.

Compared with PTT, the PDT, which employ CDs as photosensitizers (PSs) to generate reactive oxygen species (ROS) and consequently ablate cancer cells or diseased tissue, is another light-activate nanotheranostic strategy with unique advantages. Under light excitation, part of the excited-state electrons in CDs can transfer to the ambient H_2_O or dissolved oxygen and in turn lead to the type I, type II and type Ⅲ photochemical ROS production of hydroxyl radicals or singlet oxygen. On the one hand, these ROS can fervently react with the protein, lipid, polysaccharide as well as other constituting members of bacteria membrane, leading to bacterial perforation and death. On the other hand, the *in vivo* generated ROS in cells can endow the apoptosis of tumor cells, thus enabling their application for cancer therapy. Therefore, the scope of PDT treatment with CDs can be extended from tumor treatment to other application fields, such as antibacterial treatment, wound healing, inflammatory treatment, plant preservation and so on. For example, Pang *et al.* designed one kind of CDs with both intrinsic nucleolus-targeting and ROS generation capability (Figure 14A) 224. With more efficient tumor treatment induced by the ROS located within nucleolus and nucleolus from the CDs, the enhanced *in vitro* and *in vivo* PDT at a low dose of CDs and light irradiation were achieved. On the condition, various strategies such as functionalized CDs to targeted specific sub-cellular organelles were developed to further enhance the PDT effect of CDs. For example, owing to the limit therapeutic effects of oxygen-dependent PDT induced by the hypoxic tumor microenvironment and rapid consumption of oxygen in the PDT process, Jia *et al.* developed a novel CD as an in-situ tumor oxygenerator to overcome hypoxia and substantially enhance the PDT efficacy (Figure 14B) 225. In their work, researchers firstly prepared magnetofluorescent Mn-CDs with manganese(II) phthalocyanine as a precursor. With the self-assembly of DSPE-PEG, the obtained Mn-CD nanoassemblies with both NIR fluorescence and T1-weighted magnetic resonance (MR) could effectively produce ^1^O_2_ and highly catalyze H_2_O_2_ to generate oxygen, enabling them as an acidic H_2_O_2_-driven oxygenerator to improve the oxygen concentration in hypoxic solid tumors for enhanced PDT.

**Figure 15:**
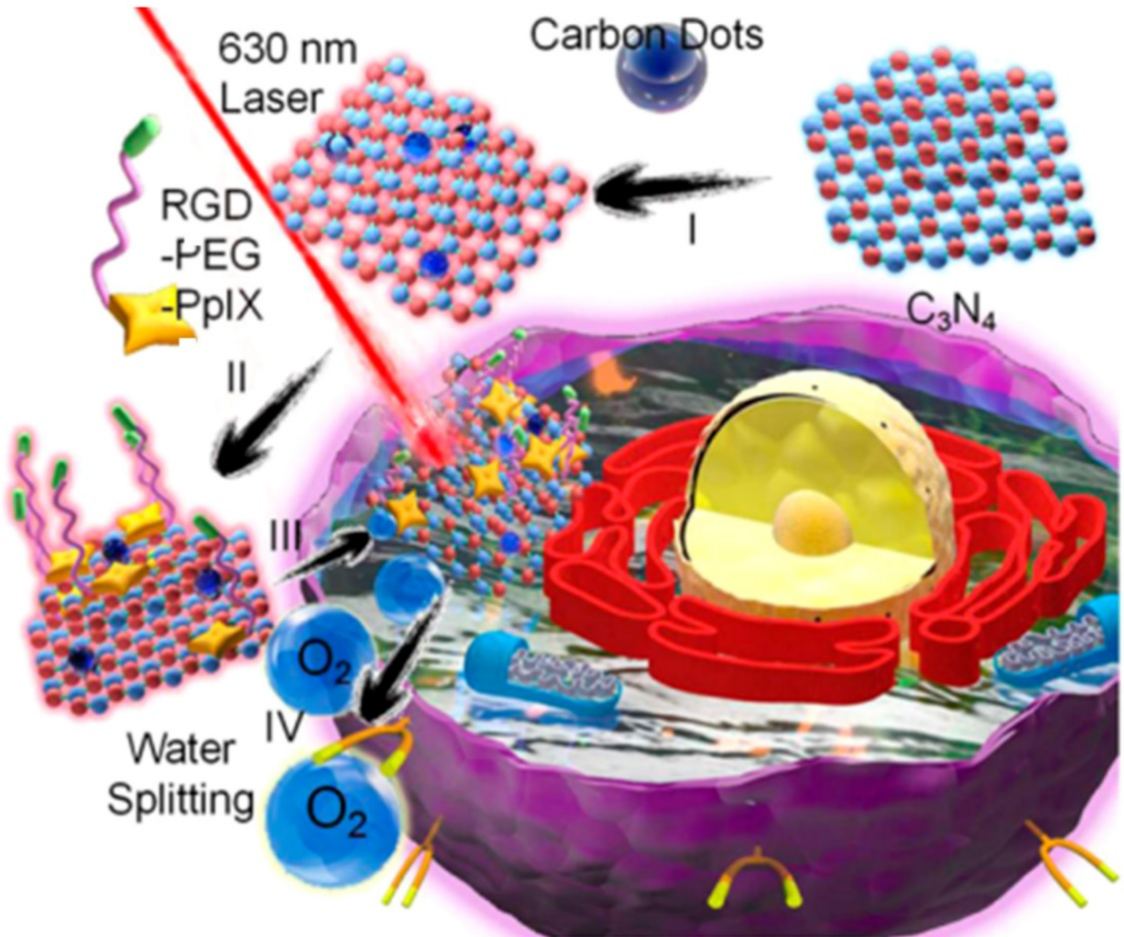
Structure of PCCN and schematic diagram of 630 nm light-driven water splitting enhanced PDT. Adapted with permission from 226, copyright 2016 American Chemical Society.

Similarly, Zheng *et al.* designed a carbon nitride (C_3_N_4_)-based multifunctional nanocomposites (PCCN) for light-driven water splitting to decrease the remarkably restriction of hypoxia in solid tumors for PDT (Figure 15) 226. In their work, researchers developed CDs-doped C_3_N_4_ to enhance absorption ranged in red region for the process of *in vivo* water splitting, and then induced a polymer with the protoporphyrin photosensitizer, polyethylene glycol segment, and targeting Arg-Gly-Asp motif into the obtained CDs-doped C_3_N_4_, successfully achieving the PCCN. With the *in vitro* study, the nanocomposites of PCCN were indicated the capability to improve the intracellular O_2_ concentration and increase the generated ROS in the hypoxic and normoxic microenvironments upon light irradiation. The experiment of cell viability approved that the PCCN could reverse the hypoxia-triggered PDT resistance, enabling a excellent growth inhibition of cancer sites in an O_2_ concentration of 1% and exhibiting superior capability to overcome the hypoxia of cancer.

**Figure 16:**
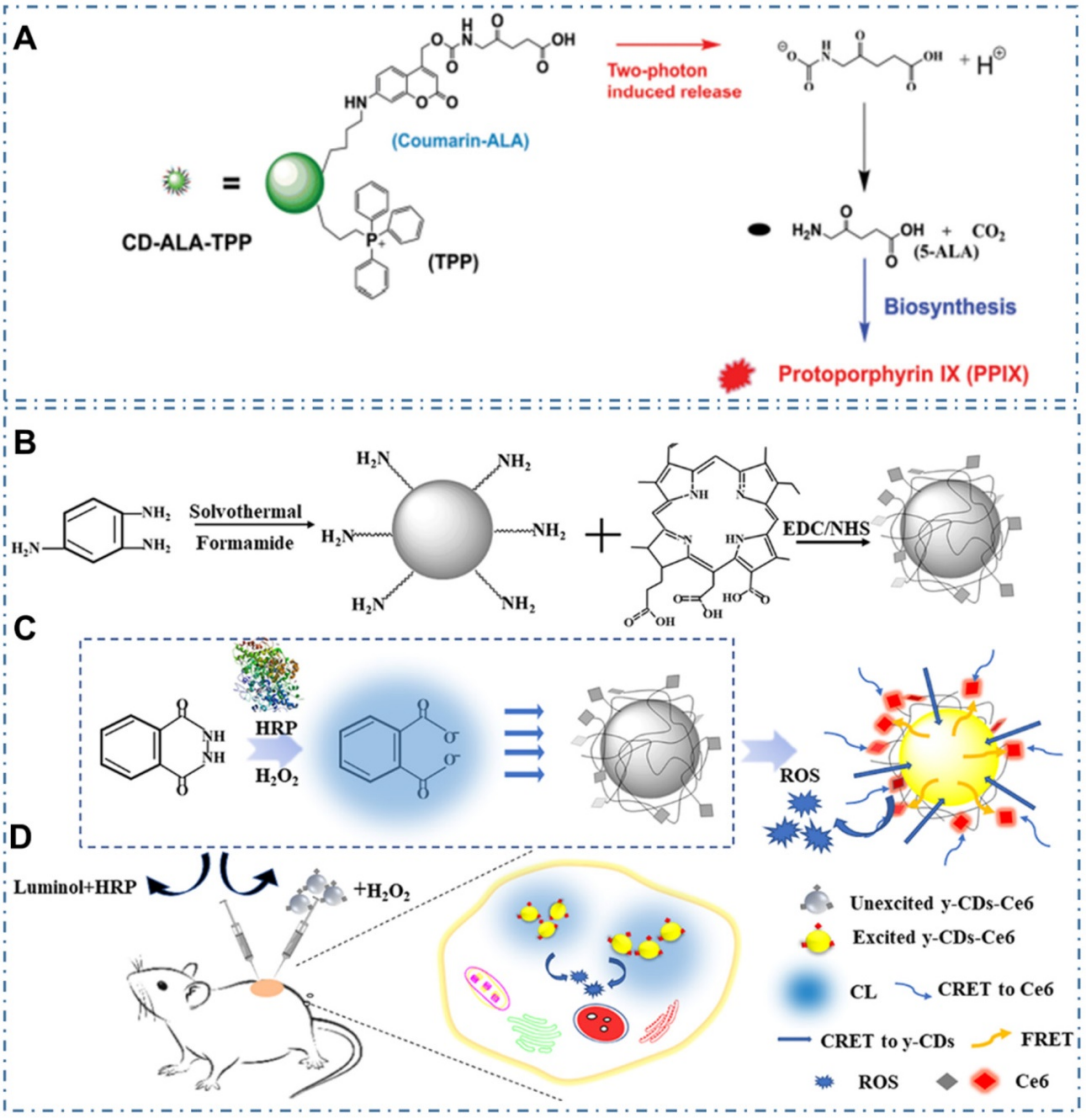
CDs-based cancer therapy by PDT with two-photon irradiation or CL. (A) Schematic illustration for the photo-triggered release of ALA from CD-ALA-TPP and the subsequent proapoptotic action on a cancer cell. Adapted with permission from 227, copyright 2015 WILEY-VCH Verlag GmbH & Co. KGaA, Weinheim. (B) Synthesis process of y-CDs and y-CDs-Ce6 conjugate. (C) Enhancement of therapeutic effect by optimization of CRET step to the PS in CL-induced y-CDs-Ce6 system. (D) Illustration of the PDT system *in vivo*. Adapted with permission from 228, copyright 2019 American Chemical Society.

In addition, due to limitation of PSs' side effects and singlet oxygen's short half-life, Wu *et al.* designed a mitochondria-targeted nanosystem to improve the PDT efficacy by releasing a bioprecursor of PSs under two-photon irradiation 227. In their work, researchers synthesized a phototriggerable coumarin derivative by linking 5-aminolevulinic acid (5-ALA, the bioprecursor) to coumarin; and then prepared the nanosystem (CD-ALA-TPP) by incorporating the coumarin derivative and mitochondria-targeting compound TPP on CDs (Figure 16A). With cellular internalization, the nanosystem exhibited selective accumulation in mitochondria; and could release 5-ALA molecules to metabolize into protoporphyrin IX in mitochondria via the biosynthesis process under one- or two-photon irradiation. With the generated singlet oxygen induced by endogenously synthesized photosensitizer under light irradiation, the CD-ALA-TPP could cause oxidant damage to mitochondria and then induce the apoptosis of the cells. Similarly, due to the major obstacles of the current PSs and the tissue penetration limit of the outer light source in PDT, Yang *et al.* developed the CL emission as an inner light source for the intracellular activation of CDs-based PDT (Figure 16B-C) 228. In their work, researchers carefully selected the nanocarriers of CDs, the inner light of CL from the reaction of luminol-H_2_O_2_-horseradish peroxidase, and the PDT agent of Ce6 to design an efficient and united system for the full use of light and enhancement of the overall PDT yield. With the observations of proliferating cell nuclear antigen (PCNA) and platelet/endothelial cell adhesion molecule-1 (PECAM-1 or CD31) results, researchers indicated the excellent performance of CL-induced y-CDs-Ce6 system in cancer therapy, providing a promising approach to achieve the selective PDT therapy (Figure 16D).

**Figure 17:**
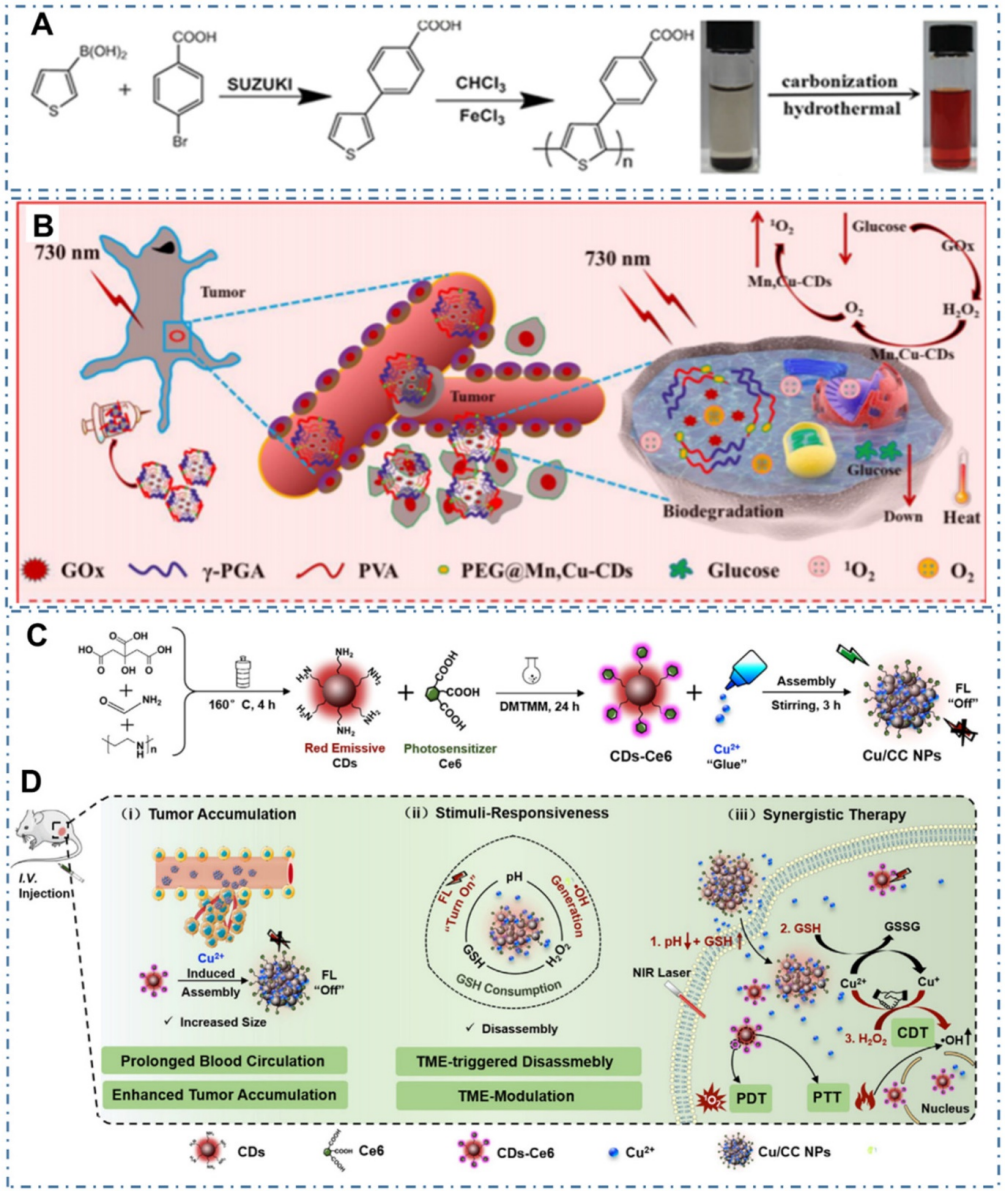
CDs-based cancer therapy by simultaneous PTT and PDT. (A) Synthetic route of PBA and CDs WITH simultaneous PTT and PDT capability by polymerization to carbonization. Adapted with permission from 119, copyright 2015 WILEY-VCH Verlag GmbH & Co. KGaA, Weinheim. (B) Schematic illustration of starving and phototherapy mediated by γ-PGA@GOx@Mn,Cu-CDs NPs. Adapted with permission from 177, copyright 2020 Elsevier Ltd. (C) Illustration of the synthesis process of Cu/CC nanoassemblies. (H) Illustration of the features for enhancing tumor accumulation, TME stimuli-responses and synergistic therapy. Adapted with permission from 229, copyright 2020 Wiley-VCH GmbH.

As CDs exhibit excellent intrinsic PTT and PDT properties, simultaneous PTT and PDT therapy in a single nanoplatform may provide the best therapeutic impact. Generally, the photosensitizers (PS) functioning as PDT agent in tumor under light can generate different ROS, thereby achieving effective treatment. Generally, the ideal PS are employed with the following characters: (1) efficient energy transfer with high quantum yield of ROS, (2) low toxicity in dark, (3) excellent photostability, (4) water solubility, and (5) broad light absorption ranging in the therapeutic window. Nevertheless, current PS from CDs are always limited by their poor water solubility, low photostability, suboptimal excitation wavelengths, or low efficiency of ROS generation. Thereupon, the combination therapy of PDT with other therapies, such as PTT, can provide the unique advantages. On the one hand, the local hypoxia in tumor can seriously limit the therapeutic effect of PDT owing to the exhaustion of tissue oxygen and fracture of tumor blood flow. Thereupon, the combination therapy can resolve the hypoxia problem to achieve enhanced anticancer efficacy. On the other hand, the combination therapy can also solve the respective limitations of common PS based on the only PDT or PTT, such as low efficacy of ROS generation, and the requirement of high photoactive power density. Thereby, considerable efforts have been exerted to develop light-triggered combination therapy. For example, Ge et al. prepared one kind of CDs with intrinsic PTT and PDT properties with polythiophene benzoic acid as carbon source (Figure 17A) 119. In their work, the obtained CDs with bright red fluorescence exhibited photodynamic capability with a singlet oxygen generation efficiency of 27% and photothermal effects with a photothermal conversion efficiency of 36.2%. On the condition, the CDs were successfully employed in the red-light-triggered photodynamic-photothermal simultaneous therapy *in vitro* and *in vivo* within the therapeutic window in the region from 600 to1000 nm. Similarly, Zhang et al. combined the action of starving therapy/PDT/PTT and checkpoint-blockade immunotherapy to improve cancer therapy 177. In detail, the immunoadjuvant nanoagents (γPGA@GOx@Mn, Cu-CDs) were designed by integrating the gamma-glutamyl transferase (GGT) enzyme-induced cellular uptake polymer-poly (γ-glutamic acid) (γ-PGA), the glucose-metabolic reaction agent-glucose oxidase (GOx), the Mn, Cu-doped CDs as PSs and self-supplied oxygenator nanodots (Figure 17B). The obtained γPGA@GOx@Mn, Cu-CDs NPs illustrated long retention time at the tumor acidic microenvironment and targeted capability for cancer cells with both photothermal and photodynamic effects under laser irradiation of 730 nm. With the endogenous generation of H_2_O_2_ caused by the nanoreactors, tumor hypoxia and further the enhancement of *in vivo* PDT were significantly relieved, enabling the improvement of therapeutic effect. Moreover, Sun et al. developed another method to design one kind of nanoplatform for fluorescence imaging and synergistic cancer therapy with tumor microenvironment stimuli-responsivity 229. With the assembling of Ce6 modified CDs (CDs-Ce6) and Cu^2+^ (Figure 17C), the as-obtained nanoassemblies (Cu/CC NPs) exhibited quenched fluorescence/PDT due to the aggregation of CDs-Ce6 and recovered fluorescence /PDT functions triggered by the stimulation of tumor microenvironment (Figure 17D). With the extra chemodynamic therapy (CDT) function through reaction with H_2_O_2_ and depletes GSH in tumors by aredox reaction introduced by Cu^2+^ in the Cu/CC NPs, the amplifier of the intracellular oxidative stress and enhanced PDT were achieved.

**Figure 18:**
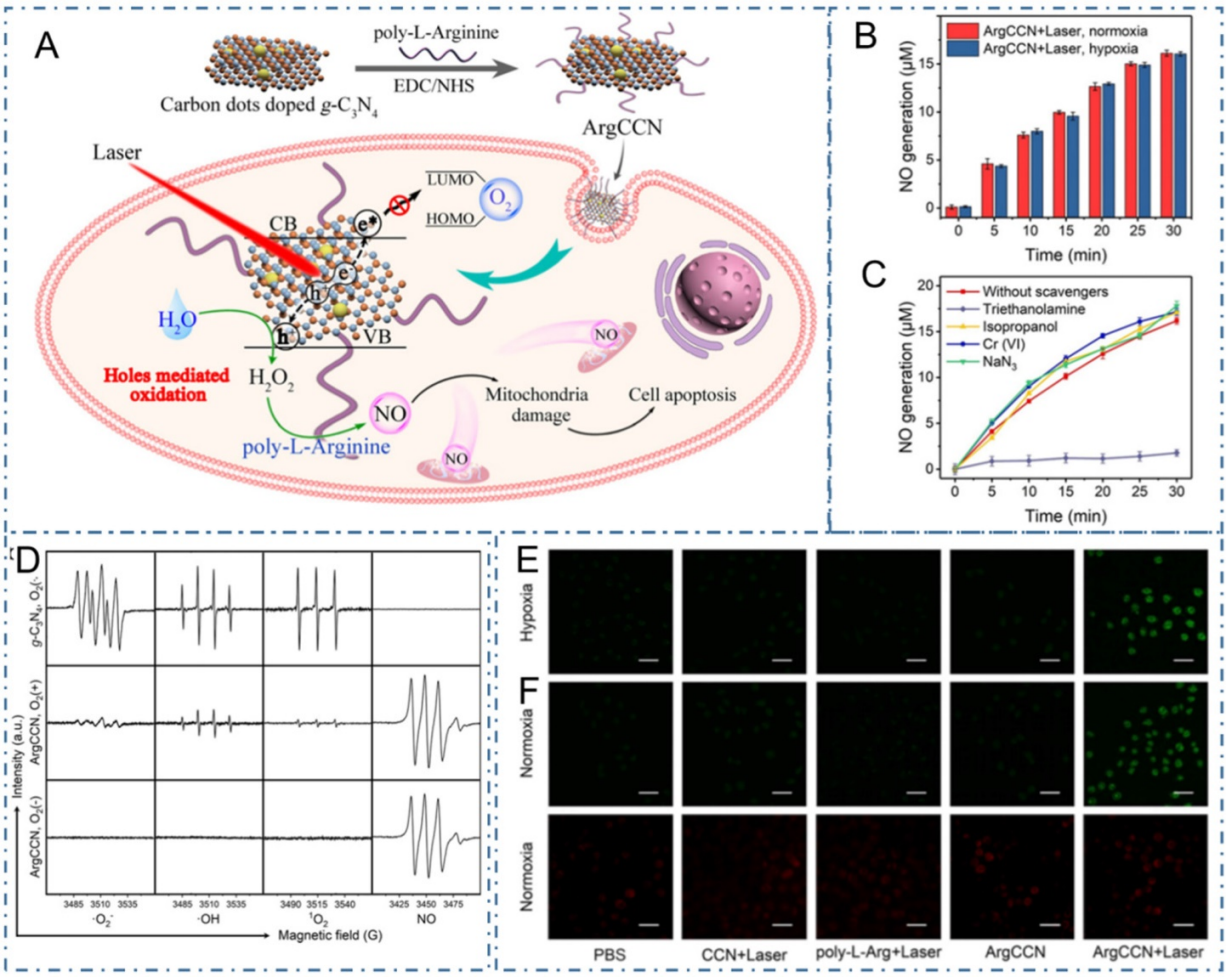
CDs-based cancer therapy by NO-based phototherapeutic. (A) The schematic diagram of microenvironment-independent NO-based phototherapeutic nanoplatform. (B) NO generation in hypoxia and normoxia; (C) ESR spectra of g-C_3_N_4_ (with O_2_) and ArgCCN (with or without O_2_) under irradiation. (D) NO generation in the presence of various scavengers. (E) CLSM images of intracellular NO generation. (F) Fluorescence images of intracellular O_2_ content probe in MCF-7 cells treated with PBS only or CCN+laser, poly-L-arginine+laser, ArgCCN and ArgCCN+laser. Adapted with permission from 231, copyright 2020 Wiley-VCH GmbH.

As well as the common PDT generated by ROS under light excitation, nitric oxide (NO) is another gaseous signal molecule with multiple physiological functions in cancer therapy 230. Due to the unsatisfactory anticancer effect of the O_2_-dependent production of NO, Fang *et al.* reported a NO-based phototherapeutic strategy mediated by photogenerated holes for hypoxic tumors (Figure 18A) 231. In their work, the phototherapeutic strategy was achieved with the NO generated by the poly-L-arginine modified CDs-doped graphitic carbon nitride nanomaterial (ArgCCN) under light excitation (Figure 18B-D). Mechanically, the holes generated by CCN could oxidize the arginine residues on poly-L-arginine to generate NO with the irradiation of 660 nm laser, thus subsequently resulting in tumor cell apoptosis. Meanwhile, regarding the large size distribution and conjugating with active tumor targeting ligands could improve the enrichment of the phototherapy nanoplatform at tumor sites (Figure 18E-F). Thereupon, the capability of ArgCCN to generate NO without O_2_ consumption could overcome the therapeutic challenges of the hypoxic microenvironment in tumor sites, paving the new approaches to cancer therapy.

### 4.3. Metabolism Effect

**Figure 19:**
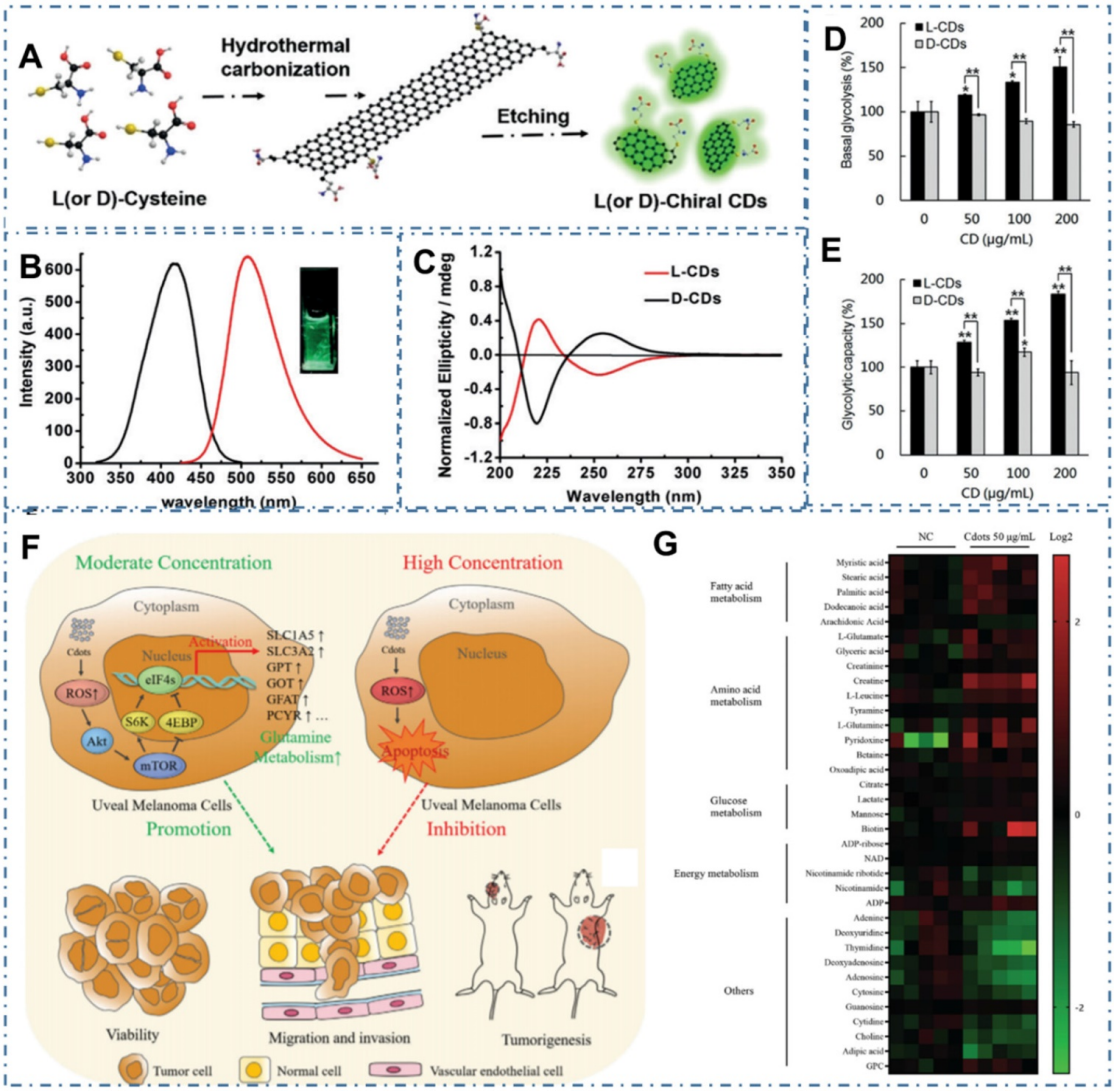
CDs-based cancer therapy by cell metabolism effect. (A) Synthesis of chiral CDs by hydrothermal treatment of chiral cysteines. (B) PL excitation and emission spectrum of the L-CDs. (C) Circular dichroism spectra of the L-and D-CDs. (D) Basal Glycolysis from the extracellular acidification rate curves. (E) glycolytic capacity. Adapted with permission from 232, copyright 2018Wiley-VCH Verlag GmbH &Co. KGaA, Weinheim. (F) Schematic of the opposing CDs-concentration-dependent effects on tumor cell progression and metastasis. (g) Heatmap depicting changes in metabolite concentration between control and 50 µg mL-1 CDs-treated Mum2B cells (p < 0.05). Adapted with permission from 233, copyright 2021 Wiley-VCH GmbH.

Despite their low systemic toxicity in mice, CDs' metabolic pathway is unclear in both normal and tumor cell growth and apoptosis. In several recent reports, researchers have observed the novel cytotoxicity of some carbon-based nanomaterials, such as carbon nanotubes, graphene, and graphene oxide, which can cause DNA and lysosomal damage and mitochondrial dysfunction, resulting in the apoptosis or necrosis. With the similar chemical structure and physical property, several kinds of CDs have been observed the similar effect on the cell metabolism. For example, Li *et al.* synthesized one kind of cysteine-based chiral optically active CDs and indicated their influences to cellular energy metabolism, which was vital for essential cellular functions 232. In their work, researchers developed a green and effective synthesis strategy for the chiral N-S-doped CDs (L-CDs and D-CDs) by hydrothermal treatment of L-or D-cysteine (Figure 19A-C). With more characterizations and experiments, the chirality-dependent enhancement of L-CDs in cellular glycolysis were observed while there were no influences on the cellular ATP levels of T24 cells (Figure 19D-E). The novel cellular energy metabolism performances indicated the potential applications of CDs in promising biomedicine for cancer therapy. In addition, Ding *et al.* observed the novel dose-dependent cytotoxicity of CDs (Figure 19F) 233. In their work, researchers demonstrated the CDs-induced dose-dependent increase of ROS levels in Uveal melanoma (UM) cell metabolism, tumorigenicity of zebrafish and nude mouse xenograft model. In practice, the effect of CDs-induced ROS could promote the growth, invasiveness and tumorigenicity of UM cells at low concentration and result in the apoptosis of UM cells of at high concentration. More experiments indicated that the CDs at 25~100 µg mL^-1^ could activate Akt/mammalian target of rapamycin (mTOR) signaling and induced glutamine metabolism, providing a cascade that promotes UM cell growth (Figure 19G). With these novel cytotoxicity capabilities in the metabolism pathway of cells, CDs could be employed one kind of potential nanomaterials for the rational design of various promising nanomedicines in cancer therapy.

### 4.4. Advanced technology

**Figure 20:**
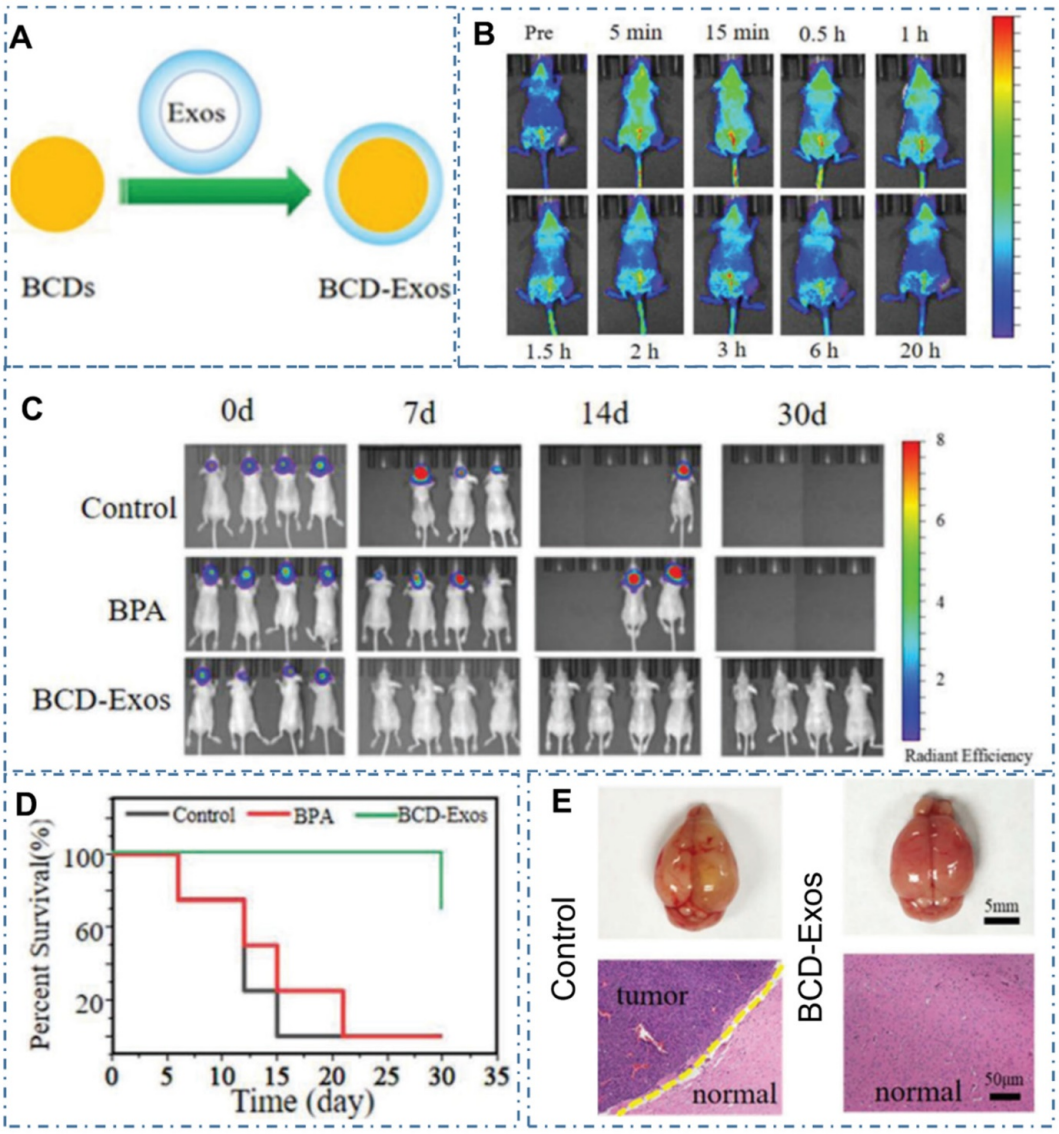
CDs-based cancer therapy by boron neutron capture therapy. (A) The preparation of BCD-Exos. (B) The ability of BCDs for bioimaging in the normal mice model. (C) The *in vivo* bioimaging of U87-Luc transplanted mice model treated with saline as the control, BPA, and BCD-Exos after BNCT. (D) Survival curves after BNCT. (E) Gross images of mice brains and microscopic images of H&E-stained tumor sections in each group. Adapted with permission from 234, copyright 2021 Wiley-VCH GmbH.

Except the above strategy to achieve cancer therapy, there are still other advanced nanotechnology to be developed with CDs for diverse cancer. For example, boron neutron capture therapy (BNCT) was a noninvasive radiation therapeutic modality for cancer therapy and Li et al. designed one kind of novel boron-containing carbon dots (BCDs) for the BNCT by tracking 10B *in vitro* and *in vivo* 234. With the encapsulation of BCDs by exosomes (Exos), researchers designed the BCD-Exos which could internalize and distribute around the nuclei of U-87-MG glioma cells (Figure 20A-B). With the ability to cross the blood-brain barrier and significant accumulation in tumor sites of the orthotopic U-87-MG glioma tumor-bearing mice model (Figure 20C), the BCD-Exos exhibited a prominent BNCT effect for the brain glioma in the mice model (Figure 20D-E). The excellent curative effect of BNCT with BCD-Exos in the application of brain glioma therapy exhibited promising potential of CDs in various neutron capture therapy.

**Figure 21:**
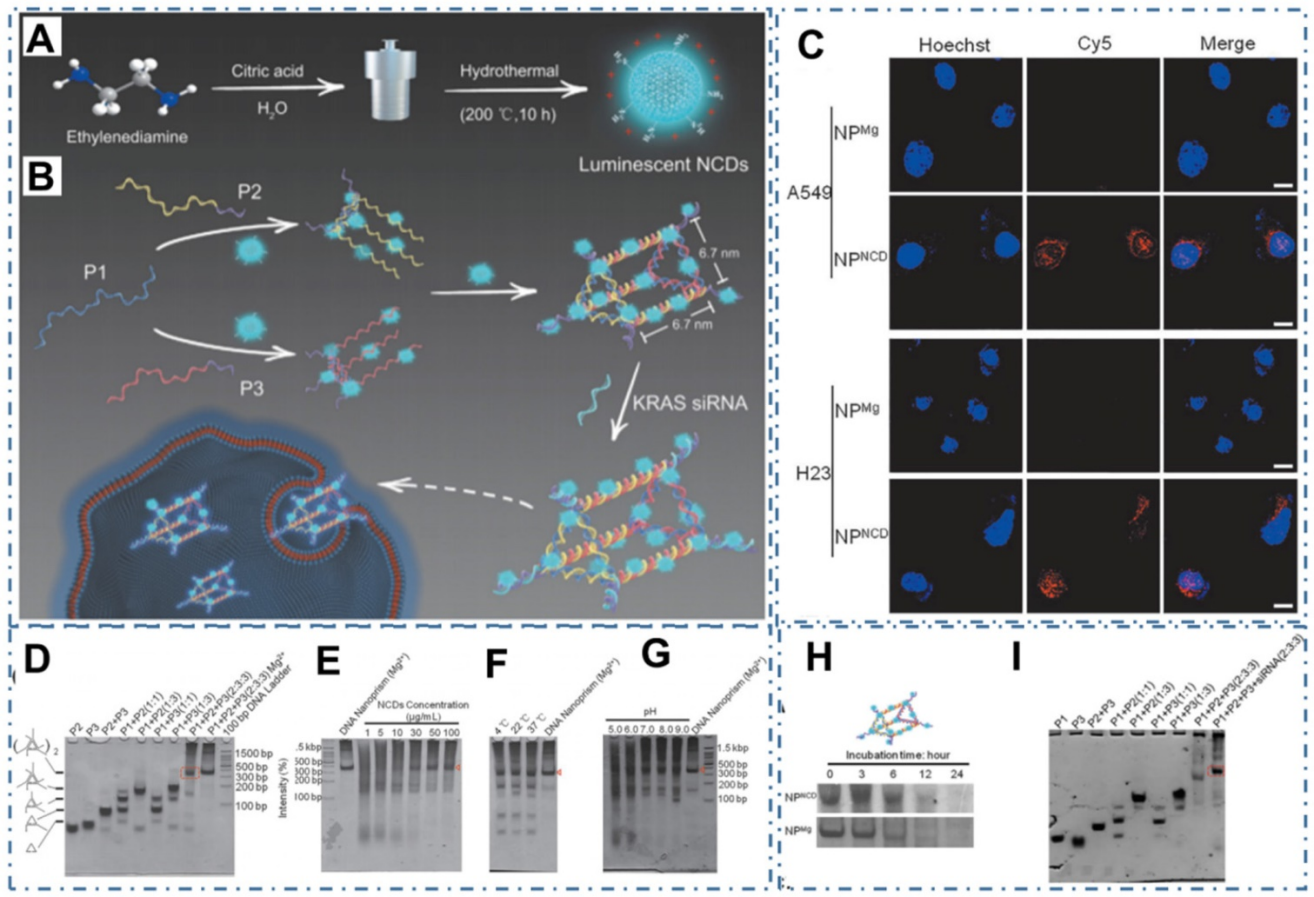
CDs-based cancer therapy by DNA nanostructure. (A) The chemical route to synthesize NCDs. (B) NCDs-assisted DNA NP self-assembly. (C) Cellular uptake evaluation of NP^NCD^ on KRAS-mutated NSCLC cell lines and CLSM imaging of NP^NCD^ internalized by A549 and H23. (D) The formation of NP^NCD^ showed by PAGE. (E) Self-assembly of NP^NCD^ at various NCDs concentration. (F) NCDs-induced isothermal DNA NP self-assembly and corresponding formation of DNA NP at different temperature. (G) PAGE analysis of NP^NCD^ formation under different pH values. (H) PAGE electrophoresis showing the serum stability of NP^NCD^ and magnesium-assembled NP (NP^Mg^). (I) PAGE analysis of the formation of CDs/NP conjugating with KRAS siRNA. Adapted with permission from 235, copyright 2020 WILEY-VCH Verlag GmbH & Co. KGaA, Weinheim.

Meanwhile, due to the scaffolds of DNA nanostructures for drug delivery, biosensing, and bioimaging by their vulnerability in physiological settings, less favorable of incorporating arbitrary guest molecules and other desirable functionalities, Wu et al. designed a DNA nanostructure self-assembly strategy mediated by nitrogen-rich CDs (NCDs) with the excellent PL and photodynamic properties and indicated the great potential of the obtained DNA/NCDs nanocomplex in anticancer therapy (Figure 21A-B) 235. In their work, researchers demonstrated the NCDs could mediate DNA nanoprism (NP^NCD^) self-assembly isothermally at a large temperature and pH range in a magnesium-free manner by polyacrylamide gel electrophoresis (PAGE) (Figure 21C-I). With excellent biocompatibility and high cellular uptake efficiency of NP^NCD^, the designed NP^NCD^ with KRAS siRNA (NP^NCDK^) was further conjugated for KRAS-mutated nonsmall cell lung cancer therapy (NSCLC), illustrating excellent gene knockdown efficiency and anticancer effect *in vitro*. These advanced technologies could greatly expand the applications of CDs in an increasing range of significant precise cancer therapy.

## 5. Discussion and Conclusion

In this paper, we have summarized and highlighted the current progress of CDs-involved nanotheranostic application in targeted bioimaging and cancer therapy. These results imply the various advantages to employ CDs as nanoplatforms to achieve targeted imaging and therapeutic functions for tumors. Through the diverse biodistribution in tumor sites and normal organism, charge and pH effect of CDs in tumor microenvironment, biomarker sensor and the self-targeting for some tumor, CDs illustrate excellent capability of *in vivo* targeted tumor cells or sites. Meanwhile, with the capability as nanocarriers for drug delivery, as agent for light-active theranostic, as interference to affect cell metabolism and as nanoplatforms for achieving advanced technology, CDs are employed to achieve precise cancer therapy. These recent progress of CDs in targeted bioimaging and cancer therapy approved the promising potential of CDs for the clinic diagnose and treatment for cancer in future.

## 6. Future prospect and outlook

In this paper, the recent progress of CDs-based nanotheranostic in targeted bioimaging and cancer therapy has been summarized. Although there have been various progresses in the nanotheranostic of CDs, there still remains various challenges to be resolved for the future biological applications.

Firstly, the structures of CDs remain to be analyzed. Compared with the molecular probes, the structures of CDs and their corresponding luminescence mechanism are not clear, thereby restricting the improvement of structure and luminescent properties for the clinic requirement.

Secondly, the metabolism of CDs in living body remains to be further analyzed. Compared with the classical medicine, the circulation of CDs in living body and organs is not clear and the interaction between CDs and living molecular is complex, leading to the limitation of CDs in clinic applications.

Thirdly, the risk of CDs for clinic therapy applications remains more studies. Though most of CDs exhibit low toxicity, there are still widespread concerns about the risk of CDs for therapy applications. On the one hand, there have been various animal studies revealing that other carbon family nanomaterials like CNTs, can be internalized by macrophages and induce inflammation and injury in the respiratory system. So far, reports of CDs for therapy applications have employed in different tissues. There still are no reports about the inflammatory response or neoplastic lesions known about whether CDs exposure may eventually affect cancer progression via possible systemic effects on non-adjacent organs. On the other hand, there have been several reports about the novel cytotoxicity in some kinds of CDs, such as their ROS-generation toxicity, dose-dependent toxicity, etc. Thereby, the risk of CDs for clinic therapy applications still needs more concerns and studies.

Fourthly, the principle and mechanism about the novel properties of CDs, such as self-targeting, metabolism effect, etc. still remain to be further analyzed and developed for more applications.

At last, more advanced nanotechnologies about CDs and their nanocomposites remain to be employed for overcoming the practical limitations in classical diagnosis and therapy.

## Figures and Tables

**Table 1:** Summary of the types of CDs and their nanocomposites with different sizes, morphology/composition, and photophysical properties.

Type	Shape	Chemical composition (elements)	Size [nm]	λ_abs_ / range [nm]	PL_max_ [nm]	PL lifetime [ns]	PL QY	PCE @λ_ex_	ROS, QY	Ref
CDs	Spherical	C, O, S	10 ± 4	400-750	640	2.3%	N/A	38.5%@671 nm	N/A	116
Supra-CDs	CD aggregates	C, O, N	12.4	600-800	N/A	N/A	N/A	53%	N/A	117
CyCD	Spherical	C, O	2.9 ± 0.5 (D) 2.8-5.0 (H)	770	820	N/A	5.7%	38.7%@808nm	N/A	118
CDs	Spherical	C, O, S	6-10 (D) 3-5 (H)	400-700	640	3.5		36.2%@808nm.	^1^O_2_, 27%	119
R-CDs	Spherical	C, O, N	4	550	640	0.68	22.9%	44%	N/A	120
NIR-PCNDs	Disk-like	C, O, N	7.8 (D) 2.1 (H)	277, 450-631	710	3.7	26.28%	N/A	N/A	121
CDs	Platelet-like	C, O, N, S, Se	20 (D) 4 (H)	526	730, 820	N/A	0.2%	58%@635nm	N/A	122
R-NBE-T-CQDs	Triangular	C, O	3.9	582	598	6.6	54%	N/A	N/A	87
CDs	Spherical	C, O, N	7.6	268, 277, 634	750	2.1	43%	N/A	N/A	123
CDs	Disk-like	C, O, N, S	2-5 (D) 0.5-2 (H)	340, 455, 605, 650	720	N/A	0.2%	59%@655 nm	N/A	39
CDs	Spherical	C, O, N	2	270-400	450 nm	N/A	20%	N/A	^1^O_2_, 0.82	124
N-O-CDs	Spherical	C, O, N	1.2-3.3 (D) 1.06 (H)	375/600-850 nm	475	16.1%	N/A	38.3%@808	N/A	125
CDs-650	Spherical	C, O, N	7 (D) 8.5 (H)	250, 365, 650	530	N/A	N/A	54.3%@655nm	N/A	126
DPP CDs	N/A	C, O, N, S	3.72 ± 0.67	530	N/A	N/A	N/A	N/A	^1^O_2_, 27.6%	127
CuCD NSs	Sheet-like	C, O, N, S, Cu	23.4	340	N/A	N/A	N/A	41.3%@808 nm	N/A	128
CuSCDB@MMT7	Spherical	N/A	222.5 ± 20.1	N/A	N/A	N/A	N/A	39.7%@808nm	N/A	129
BCCGH	Sphere-like	C, O, N, S, Cu, Gd	7.9	605/ 640-700	655	N/A	N/A	68.4%@808nm	N/A	130
CDs	Spherical	C, O, N	5.6	226, 280, 615	648, 685	20	34%	N/A	N/A	131
CDs	Spherical	C, O, N	4-5	560	630, 670	N/A	25%	N/A	N/A	132
NIR-CDs	Disk-like	C, O, N	4 (D) 0.4-2 (H)	619, 720	770	0.561	11%	N/A	N/A	133
CNDs	Spherical	C, O, N	2.5	540	600-900	3.3	56%	N/A	N/A	90
Ce6-RCDs	Spherical	C, N, O	3.7	405, 640	653	N/A	N/A	46%@671 nm	N/A	134
Ni-pPCDs	Spherical	C, N, O, Ni	2.9 ± 0.5	204, 243, 286, 510	605	N/A	45.6%	N/A	N/A	135
NIR-CD/MoS_2_	Sheet	C, O, N, S, Mo	100	450	N/A	N/A	N/A	78.2% @808 nm	N/A	136
DA@N-CDs(Mn) NPs	Spherical	N/A	3.1	530	620 nm	4.5%	N/A	28.2%@808 nm	N/A	137
CQDs	Disk-like	C, O, N	2.42	628	665	2.49	47%	N/A	N/A	138
C-CD /TiO_2_	spherical	C, O, Ti	244.6	285, 400	N/A	N/A	N/A	9.54%@808nm	N/A	139
CDs	spherical	C, N, O, S	4.4	315, 355/ 320-750	930	4.59, 13.08	∼8.0%	54.7%@808nm	N/A	140
SCDs	spherical	C, O, N	20	337, 600	N/A	N/A	N/A	41.7%@808 nm	N/A	141
CDs	Spherical	C, O, N, F	2.6	556, 624, 715, 847	658, 777	6.8	10	N/A	N/A	142
CDs	Spherical	C, O, N	4	295, 395, 580	642	5.9	N/A	N/A	N/A	143
CDs	Spherical	C, O, N	4-6	339	449	2.56	0.39%	N/A	O_2_^-^, 1.08%	144
Se/N-CDs	Spherical	C, O, N, Se	3.6 ± 0.6	317,550	607	N/A	3.70%	N/A	^1^O_2_, 10.6%	145
CCOF @PEG	Spherical	N/A	235	200-900	650	N/A	N/A	N/A	N/A	146
NCDs	Spherical	C, O, N	4.1 ± 1.6 (D) 2.3 ± 0.8 (H)	610	623			77.6%@660	^1^O_2_, 37%	147
S-CDs	Sphere-like	C, O, S,	3.2 (H) 2.0 (H)	300, 360/ 400-900	440	N/A	N/A	55.4%@808 nm	N/A	148
Ni-CDs	Spherical	C, O, N, Ni	4.6 (D) 1.9 (H)	1002/ 750-1350	N/A	N/A	N/A	76.1%@1064 nm	N/A	149
S, N-CDs	Spherical	C. N. O, S	9	560/ 600-700	630	N/A	12.4%	34.4% @808nm	^1^O_2_, 27%	150
TP-CDs	Quasi-spherical	C, O, N	4.0 ± 1.1	274/400-600	605	N/A	N/A	N/A	N/A	151
RGQDs	Spherical	C, O	3.54	230	532, 950	N/A	6%	N/A	N/A	152
CDs	spherical	C, O,	3-4	310, 365	502	N/A	N/A	N/A	^1^O_2_, 0.51	153
f-CDAs		C, O, N	12-22 (D) 7-13 (H)	550-700	675	2.3	15.6%	26.1%@655 nm	N/A	154
FA-CD/PPy-NPs	spherical	C. N. O	6.08 ± 2.07	260/ 700-1000 nm	520	N/A	20.39 ± 1.80 %	40.80 ± 1.54% @808nm	N/A	155
NIR-II-CD/BP Hybrids	Sheet-like	C, N, O, P	100-200 (D) 2.0 (H)	420	480	N/A	N/A	61.4%@1064 nm 77.3%@808 nm	N/A	156
anti-EpCAM@ PDA-CDs@Pt(IV)	Spherical	C, N, O, Pt	1.7	275, 360	454	N/A	∼22.5%	39.1%@808 nm	N/A	157

Abbreviations: D—diameter; H—height; λ_abs_—Maximal absorption wavelength; λ_ex_—excitation wavelength; λ_em_—emission wavelength; PCE@ λ_ex_—photothermal conversion efficiency @ corresponding excitation wavelength; and ROS, QY—reactive oxygen species, quantum yield.

**Table 2:** Summary of the types of CDs and their toxicity and drug loading capacity.

Type	Size of CDs	Size of nanocarriers	Surface Engineering	Drug	DLC, (wt%)	Cell lines, Animal models	Observation	Ref
Qucbl-CDs	5-7 nm	60-80 nm	Covalently anchored	Qucbl	N/A	Hela	Qucbl-CDs: 0-50 μM, cellviability >90%	203
DOX-CDs	6.8±_1.3 (D) 8 (H)	N/A	Physisorbed through interactions such as π-π stacking, hydrophobic and van der Waals interactions	DOX	6.0	A549	DOX-CDs: 400 μg mL^-1^, no growth inhibition	204
CA-CD	N/A	220 ± 25.99 um	Hydrogen bonding	b-TC	77	N/A	N/A	205
CDs-Oxa	2.28±0.42 nm (D) 0.34-1.4 nm (H)	2.71 ± 0.43 nm (D) 2.5-4.2 nm (H)	Condensation reaction between the amino groups and the carboxyl group	Oxa(IV)-COOH	4.2	L929; HepG2	CDs: 0.5 mg mL^-1^, survival rates > 75%; CD-Oxa: cytotoxic as oxaliplatin(II) (IC_50_ = 3.4 µg mL^-1^)	206
CDs-Pt(IV)@PEG-(PAH/DMMA	7 nm	125 nm	Intriguing charge conversion to a cationic polymer	Pt(IV)	6.7	A2780	CDs: 3.125-400 μg mL^-1^, no toxicity (A2780); CDs-Pt(IV)@PEG-(PAH/SA): 0.18-11.44 μM, no toxicity to cancer cells at pH 6.8 or 7.4	207
CDsRGD-Pt(IV)-PEG	5-8 nm	31 nm	Intriguing charge conversion to a cationic polymer	Pt(IV)	4.1	MDA-MB-231, MCF-7 cells	CDs: 200 μg mL^-1^, negligible toxicity, CDs-RGD-Pt(IV)-PEG: at pH 6.8, much higher cytotoxicity	208
PNHCDs-DOX	5.8±0.1 nm (D) 3.8 nm (H)	N/A	Interactions such as electrostatic attraction, π-π stacking, van der Waals force, and hydrophobic interaction	DOX	35.43	HepG2, SiHa, MCF-7	PNHCDs: no affect to cell survival; PNHCDs-DOX: 400 μg mL^-1^, 65-70%	209
CD-DOX conjugates	2-6 nm	N/A	Amines bind with the carboxylic acid via electrostatic interactions or hydrogen bonding.	DOX	N/A	HepG2 cells HL-7702 cells	CD: 1.5625-100 mg mL^-1^, no influence; CD-DOX: dose-dependent death	210
P-CDs/HA-Dox	1.4-3.2 nm	15-30 nm	Electrostatic self-assembly	DOX	6.3	HeLa, NIH-3T3	PCDs/HA-Dox: 50 μg/mL, 90% of NIH-3T3 cells survive	198
IL-OCDs/Cur	7.2 nm	N/A	Hydrophobic interaction	Cur	69.2	HeLa cells.	IL-HCDs: 50 μg mL-1, cell viability of >80%,	211
DOX@ACD	119±207 nm	131 ± 3.7 nm	Amphiphilic interaction	DOX	14.2 ± 0.003 wt%	A549	ACD@DOX: higher cell viability at low concentrations and lower viability at higher than DOX.	212
FA-Gd@CQDs/DOX	4.0 ± 0.7 nm	N/A	π-π stacking and hydrophobic interactions.	DOX	74.5 ± 3.96	HeLa, HepG2, and HeLung cell	FA-Gd@CQDs: low cytotoxicity, FA-Gd@CQDs/DOX: greater cell growth inhibition toward HeLa cells than DOX	213
pCBMA(CD-D/DOX)	3 nm	183 ± 27 nm (D) 200 nm (H)	Electrostatic interactions and π-π stacking	DOX·HCl	96.9	4T1 and HepG2 cells	CDs: 0.01-5 μg/mL^-1^, survival rates ~100%; pCBMA(CD-D/DOX): at low DOX dose (0.01, 0.1 μg/mL), 4T1 cells incubation rates ~100%	214
CDs-epi	1.5 (D) 1.7(H)	2.6 (D) 3.5 (H)	Conjugated	Epirubicin	N/A	SJGBM2, CHLA200, CHLA266, U87	CDs-epi: 10 μM, 17-30% survival rates	215
p(CAT2-CD-BA1)	2.72 nm	3.79 nm	Noninvasive adsorption	DOX	84.28	HeLa	p(CAT2-CD-BA1): 100 μg mL^-1^, cell viability unchanged	216
CD-PEI-DOX	2-8 nm (D), 2-10 nm (H)	222.5 ± 20.1 nm	Electrostatic interactions	DOX	35.88	L02, MHCC-97L, Hep3B	CD-PEI-DOX: (<10 μg mL^-1^, effective inhibition, far less cytotoxic to L02 than to cancer cells	217
DS-NA	5 nm	7235 ± 2.9 nm	Hydrophobicity interaction	DOX	23.5	MDA-MB-435S, 4T1	DS-NA: higher cytotoxicity to MDA-MB-435S cells (IC_50_ = 4.841 μg mL^-1^) than 4T1 cells (IC_50_=16.08 μg mL^-1^)	218
APCDs@Fe/DOX-LOS	9.4±0.62 nm	106 ± 1.5 nm	π-π stacking interaction with large p-conjugated structure of APCDs	DOX	30.3 ± 1.3	4T1 tumorbearing mice	Blood biochemical parameters: levels remained normal ranges, H&E staining: no noticeable morphological damage or inflammatory injury	219

Abbreviations: D—diameter; H—height; DLC—Drug loading capability
